# Emergent Spinning and Orbital Motion in Clustered Wind‐Assisted Flyers

**DOI:** 10.1002/advs.76560

**Published:** 2026-07-11

**Authors:** Bingnan Zhou, Rahul Chand, Fahad Ahmed Korai, Jianfeng Yang, Valtteri Savonen, Fan Liu, Qi Yang, Hao Zeng

**Affiliations:** ^1^ Light Robots, Faculty of Engineering and Natural Sciences Tampere University Tampere Finland; ^2^ Key Lab of Rubber‐Plastics Ministry of Education/Shandong Provincial Key Lab of Rubber‐Plastics School of Polymer Science and Engineering Qingdao University of Science & Technology Qingdao China

**Keywords:** clustering, flying robot, liquid crystalline elastomer, vortex, wind dispersal

## Abstract

Nature offers a rich diversity of wind‐assisted flyers, ranging from snowflakes to dandelion seeds and fungal spores. Despite their differences in size, material, and aerodynamic regime, these systems share a common principle: when individual flyers aggregate into clusters, the resulting geometric asymmetry gives rise to emergent behaviors, e.g., spinning, orbital motion, and extended gliding. This cluster‐induced aerodynamic asymmetry represents a general mechanism in passive aerial dispersers, yet it remains poorly understood and rarely studied in a controlled manner. Here, we present a physical model of a wind‐assisted flyer capable of cluster‐induced spinning. The flyers are designed with porous and laser‐cut from office paper. A single flyer exhibits airborne stability due to the formation of vortex rings downstream. When two flyers form a cluster, the clustering handedness determines the spinning velocity. Computational simulations reveal that the torque difference induced by structural asymmetry plays a critical role in determining the mid‐air motion. With experiments, we demonstrate peak autorotation efficiency at 5° angular mismatch between two flyers. Furthermore, we explore mid‐air self‐assembly of flyers driven by aerodynamic interactions, during which dynamic clustering and de‐clustering behaviors are observed, yielding diverse spinning velocities. These findings offer new possibilities for programmable mid‐air clustering motion.

## Graphical abstract

Inspired by natural snowflake geometries, paper‐cut flyers exhibit geometry‐defined spinning and orbital motions during cluster formation in a wind tunnel. Collective flights of multiple paperflakes reveal dynamic clustering, fragmentation, and continuously evolving spin behaviors.

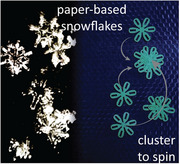

## 1 Introduction

A wide range of structurally ingenious passive flight systems exist in nature, especially in the dispersal of plant seeds or spores [1, 2, 3]. These biological miniature systems are typically categorized as winged or parachute‐like structures, which leverage interactions between their morphological features and ambient airflow to achieve stable, prolonged gliding or dispersal over long distances [4, 5, 6]. For instance, maple seeds generate leading‐edge vortices (LEVs) through their samara wing structure, allowing them to spin rapidly and extend their time aloft [7, 8]; dandelion seeds, on the other hand, make use of a parachute‐like pappus structure to form a stable low‐pressure vortex ring underneath, enabling ultra‐slow descent and wide‐area dispersal [9, 10, 11]. A few key design principles that researchers drew from these natural features for aerodynamic optimization are: lightweight and low density [12, 13, 14], high stability through a centrally symmetric architecture [8, 10], and intentional asymmetry to induce disrupted flow for spinning or tumbling [15, 16].

Among the diverse dances of mid‐air flyers, spinning is one of the most typical phenomena. It often arises from the asymmetry near the leading edge [3, 15, 17]. Taking advantage of the merit of leading‐edge vortex aerodynamics, researchers have designed glide‐capable structures using smart materials that deform in response to external stimuli (e.g., heat, light, electricity), allowing in‐flight regulation of spinning behavior. For example, research has progressed from employing shape memory polymers for morphable wing surfaces [7], to using light‐responsive materials for controlling the rotational dynamics of artificial single‐wing seeds [9], and more recently, to implementing photochemical mechanisms with liquid crystal elastomers (LCEs) [15]. These studies collectively demonstrate the feasibility of using stimulus‐responsive materials to actively modulate passive flight behavior without significantly altering the mass or global symmetry, thereby extending the function boundaries of traditional flyers, which are often made of purely passive materials [18, 19]. In addition, the emergence of miniature flexible sensor technology has enabled the integration of environmental sensors into bio‐inspired gliders, allowing real‐time monitoring of environmental parameters of temperature, humidity, pH levels, etc [4, 5, 8, 16, 17, 20]. These functionalized artificial seeds retain the aerodynamic advantages of their natural counterparts while significantly expanding their potential for cross‐disciplinary applications.

However, current research on the role of clustered structures in wind‐dispersed flight systems remains very limited. While active clustering, as seen in migratory bird flocks, is known to enhance flight efficiency [21, 22, 23, 24], passive clustering in plant or insect systems – driven by aerodynamic interactions or gravity – can also induce complex collective motion [25, 26]. This collective phenomenon in natural species leads us to ask scientific questions: Can man‐made structures spontaneously form in‐air clusters? Can these clusters mimic the complex mid‐air motions observed in natural systems, i.e., exhibiting emergent behaviors that go beyond the capacity of any individual flyer?

Trying to answer these questions, we introduce a wind‐assisted dispersal model system based on laser‐cut paper structures that serve as geometric analogs of snowflakes. When laser cut and levitated in a steady wind tunnel environment, these structures transition from non‐rotating individuals, to spinning and orbiting pairs when clustered, with dynamics exquisitely sensitive to their relative alignment and connection spacing. Moreover, multiple structures undergo dynamic clustering and de‐clustering in mid‐air as a result of complex inter‐structure vortex interactions.

## 2 Results

### 2.1 Artificial Snowflakes

**FIGURE 1:**
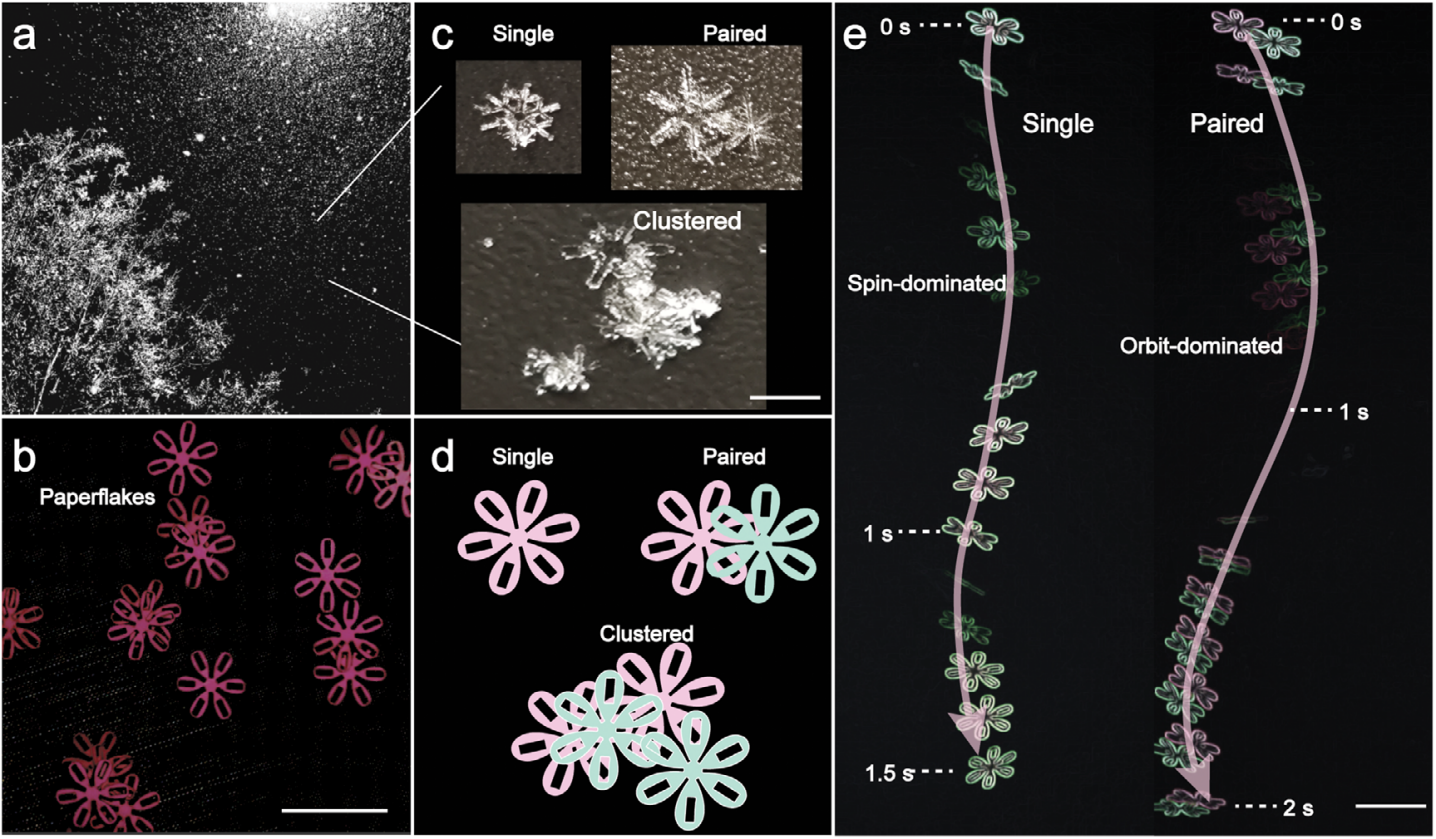
Snowflakes and paperflakes. (a) Photograph of the falling of snowflakes. The photo was taken at typical Finnish winter night upon the illumination of a streetlamp. (b) Paperflakes flowing and clustering inside a stable wind field venting from below. (c) Photographs for representative morphology of natural snowflakes. Scale bar: 2mm. (d) Schematic illustration of paired paperflakes and possible clustered configurations. Scale bar: 30mm. (e) Superimposed images and trajectories for single and paired paperflakes free falling inside the still air. Scale bar: 30 mm.

The lightness of snowflakes primarily arises from their porous crystalline structure and the high air resistance, enabling them to descend slowly through the atmosphere. During the Finnish winter season, we often observe that, descending snowflakes in a non‐wind condition, there diverse dances in the mid‐air appear, i.e., lateral drifting, zigzag motion, self‐rotation, orbital motion, and irregular tumbling (Figure 1a) [27, 28]. Such elegant yet complex aerial trajectories are due to the complicated snowflake geometries result from the growth of irregular snow clusters. Inspired by snowflake clusters, we developed laser‐cut artificial snowflakes (paperflakes) with sixfold central symmetry (Figure 1b). Structural robustness and high porosity were achieved by hollowing the center of each lobe, a strategy that avoids the use of deformation‐prone, fine substructures in the office paper material. As shown in Movie S1, after spreading tens of paperflakes into the wind tunnel, they flow in the middle of the air, cluster into groups that start to show different kinds of locomotion activity, which are analogous to the complex mid‐air dances of snowflakes. Three quick conclusions can be drawn from the observation of the movie. First, a single paperflake exhibits good airborne stability in the wind with minimal rotation. Second, interactions in the surrounding airflow enable them to form dynamic and unstable clusters, which may be disrupted by collisions. Third, pronounced self‐rotation and orbital motion are observed from the in‐air clusters. This study is to understand the aerodynamics of the paperflake model and explore the way to control its clustering performance in air.

It is known that natural snowflake crystals, due to the regular arrangement of water molecules formed by hydrogen bonding during crystallization [29, 30], tend to form highly axisymmetric structures (Figure 1c). For a simple crystal grown at the perfect form, its falling trajectory should be a straight vertical descent inside a steady air condition, without lateral motion or rotation [27]. In reality, snowflakes grow into clusters, forming asymmetric structures macroscopically (Figure 1c). Coincidentally, we observed similar clustering in our paperflakes (Figure 1d). Paperflakes readily aggregate once they approach each other because the low‐pressure wake region behind a falling flake draws neighboring flakes into close proximity [31]. When two flakes are joining closely and performed the free‐falling experiment inside steady air condition, a spin‐dominated descent is observed. When two flakes are connected at a relatively large distance, the cluster spins accompanied with orbital motion (Figure 1e). Figures S1 and S2 present the top‐view trajectories and the corresponding height–time (*t*) relationships of the falling process for these paired paperflakes. The 2D design of the paperflake, along with the parameters used for fabrication, is shown in Figure S3.

### 2.2 Aerodynamics of Individual Paperflake

**FIGURE 2:**
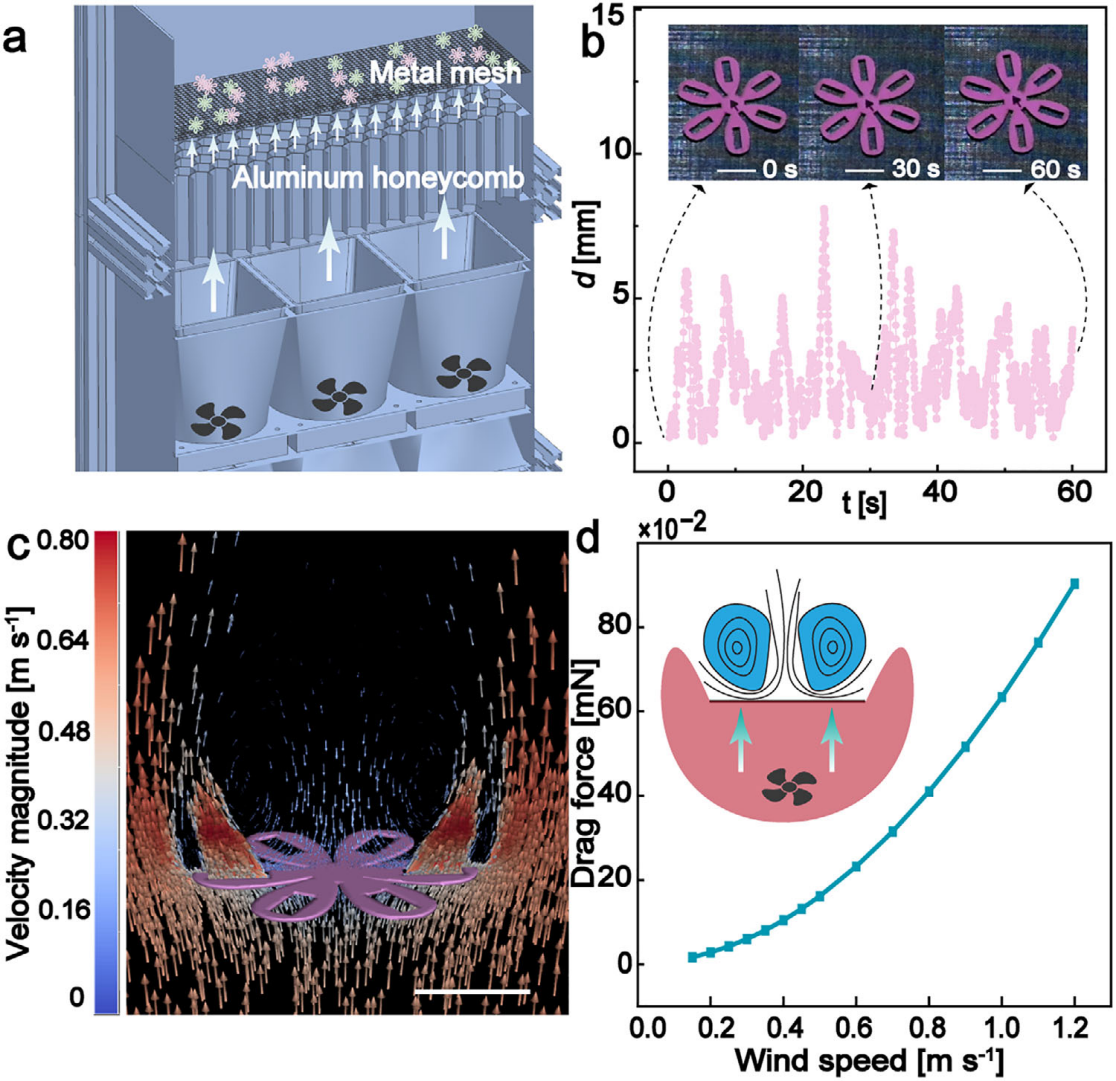
Aerodynamics of single paperflake. (a) Internal structure of the wind tunnel. (b) Time‐lapse images showing the posture of a paperflake at 0, 30, and 60 s and its displacement over time (*d* is the displacement of the centroid). (c) Simulated airflow field around a fixed paperflake inside a constant wind flow (0.3 m s^−1^). (d) CFD simulations of the drag forces acting on paperflakes. Scale bars are 10 mm.

The wind tunnel setup used in this study is schematically shown in Figure 2a. The airflow source consists of several parallel‐connected fans. A honeycomb panel is placed on top to straighten the stream, and a metal mesh is positioned between the fans and the test section to prevent accidental sample drop into the fans. The spatial fluctuation of the vertical airflow velocity inside the wind tunnel was experimentally characterized by measuring the vertical velocity component at five different heights above the honeycomb mesh (0, 10, 140, 280, and 420 mm). At each height, velocities were recorded over a 5 × 5 grid, resulting in a total of 125 sampling points. The normalized spatial velocity fluctuation was estimated as $\;I\;\left(x,y,z\right)=\frac{u_{x,y,z}-\bar{u}}{\bar{u}}\;,$where *u*_*x*,*y*, *z*_is the measured vertical velocity at the position of [*x*, *y*, *z*], $\bar{u}$ is the overall mean vertical velocity. The spatial distribution of *I*(*x*, *y*, *z*) is presented in Figure S4. Within this stable airflow, paperflakes are able to maintain prolonged equilibrium hovering, resulting from the counterbalance between the vertical airflow, gravity, and the vortices that accompany the flow. As shown in Figure 2b, a single paperflake remains suspended in mid‐air with minimal lateral displacement (a maximum drift of ∼7 mm, about one‐quarter of its diameter) and a change in orientation (<30°) over a period of 1 min. The time‐dependent variation of the rotation angle *γ* of a single paperflake in the wind tunnel (Figure S5) further quantified this orientational stability.

To further elucidate this phenomenon, Computational Fluid Dynamics (CFD) simulations were conducted to resolve the pressure distribution and airflow around the paperflake (see Supporting Note). Details of CFD simulation result are provided in the Supporting Note. The flow field presentations with iso‐surfaces of vorticity magnitude and pressure contour plots reveal the structure of the vortices generated downstream of the paperflake. The results suggest that the non‐vertical components of the velocity field are symmetrically distributed in the two‐dimensional plane and mutually cancel out, thereby suppressing the onset of rotation and lateral drift (Figure 2c). A detailed description of the simulation model setup and the Transition SST turbulence model is given in Note S2.1. The three‐dimensional pressure and velocity fields around a single paperflake are illustrated in Figures S15 and S16.

The CFD simulations further show that a single paperflake behaves aerodynamically like a flat plate, generating a region of lower pressure on its leeward side. Driven by centerline velocity gradients and shear layer instabilities [32], vortex rings are generated above its surface (Figure 2c). When the incoming vertical airflow impinges on the windward surface, the local velocity decreases and approaches a stagnation region. This deceleration leads to an increase in pressure, forming a relatively high‐pressure region on the windward side. This stagnation establishes a pressure difference between the windward and leeward sides, which contributes to the overall form drag [33]. Meanwhile, flow separation occurs near the edges of the paperflake, generating a wake region on the leeward side that is associated with vortex formation and reduced pressure. The pressure difference between the high‐pressure region on the windward surface and the low‐pressure wake therefore produces a net aerodynamic drag force. In the present configuration, this pressure‐dominated drag mechanism provides the dominant contribution required to balance the weight of the paperflake. Due to the extremely small thickness of the structure, the shear stress acting normal to the plane could be neglected. Using CFD simulations, we quantified the drag forces acting on paperflakes as the wind speed increased from 0.15 to 1.2 m s^−1^. The drag forces increase from 0.02 to 0.91 mN (Figure 2d). Such a trend suggests that the intrinsic geometry of the paperflake enables a robust generation of drag‐producing vortices under varying flow conditions, thereby stabilizing its aerodynamic response.

### 2.3 Cluster‐Induced Rotation

**FIGURE 3:**
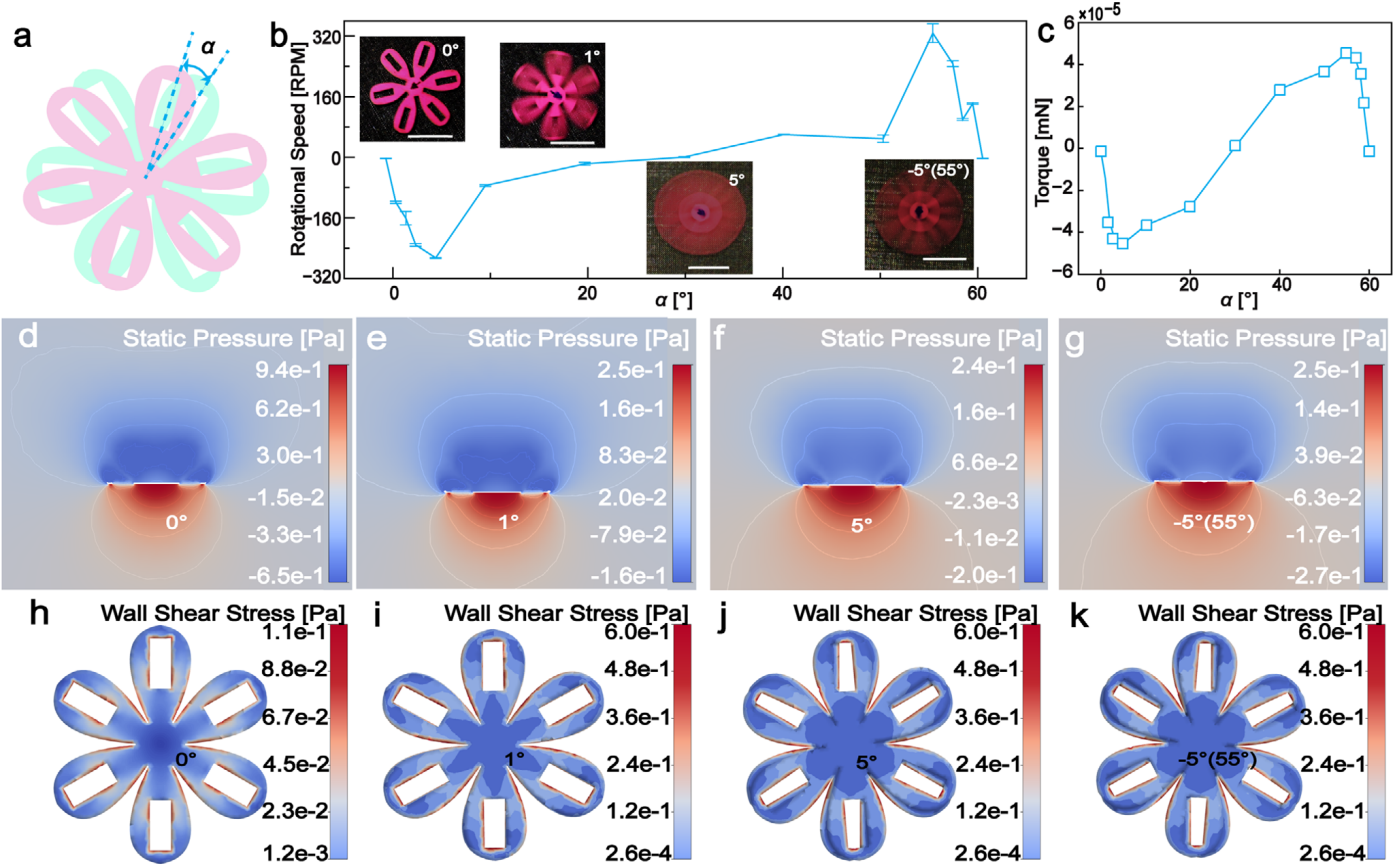
Cluster‐determined spinning. (a) Schematic drawing of a paperflake pair at zero separation distance. *α *is the relative angle representing the degree of mismatching between two paperflakes’ orientations. (b) Measured rotational speed upon the change of *α*. Inserts show snapshots of the structure at *α* = 0°, 1°, –1°, and 10°. Scale bars: 10 mm. The error bars are displayed as mean values ± standard deviation (*n*   =   3). The same sample was measured repeatedly. (c) CFD simulated torque on paperflake pair as a function of *α*. (d–g) CFD simulated pressure distributions around the paperflake pairs with 0, 1, 5 and −5° relative angle. (h–k) CFD simulated shear stress distributions on the paperflake pairs with 0, 1, 5 and −5° relative angle.

The reduced pressure upon the paperflake triggers the stacking of multiple structures inside the wind flow. The resulting clusters possess irregular shapes, breaking geometric symmetry of individual ones and exhibiting emergent aerodynamic behaviors (Movie S1). Here, we study the simplest clustering model – a pair of paperflakes. The specific form of the paperflake pair is characterized by two geometrical parameters, the relative angle (*α*) in alignment and the center‐to‐center separation distance (*L*). The former describes the difference in orientation between the flakes, while the latter specifies the distance between their centers. In the following, the details of the influence of different *α* (Figure 3a) on the mid‐air locomotion are investigated.

The paired paperflakes remain stationary at *α* = 0° and 60° owing to geometric symmetry. Their steady‐state rotational speeds were measured at *α* = 0°, 1°, 2°, 3°, 5°, 10°, 20°, 30°, 40°, 50°, 55°, 57°, 58°, and 59°, as illustrated by representative snapshots in Figure 3b. As shown in Figure 3c, CFD simulations of the net aerodynamic torque as a function of *α* exhibit consistency with the experimental results. Two pronounced peaks (>280 rpm) are observed at approximately *α* ≈ 5°and *α* ≈ 55° (equivalent to −5°) where the torque magnitude also reaches its maxima. In contrast, the torque vanishes at *α* = 0° (60°) and *α* = 30°, consistent with the absence of rotation under these symmetric configurations. The CFD‐predicted pressure distributions on the central cross‐section (Figure 3d–g) show that symmetric configurations produce nearly identical pressure fields, resulting in zero net torque. At small angular mismatches, only weak pressure asymmetry is observed, indicating that pressure difference alone plays a limited role in driving rotation. The wall‐shear‐stress distributions on the paperflake surfaces (Figure 3h–k) exhibit clear asymmetry even at small angles, providing a dominant contribution to the aerodynamic torque. Correspondingly, as *α* increases from 1° to 5°, the shear‐stress contrast becomes significantly more pronounced. Additional insight is provided by the iso‐surfaces of vorticity magnitude (Figures S14a–d), which reveal substantial changes in the wake structure even for small angular mismatches. The vortical structures expand and become more spatially extended with increasing *α*, further supporting the strong sensitivity of the flow field to geometric perturbations. As *α* increases further, the rotational speed decreases and falls to zero at *α* = 30°, where a higher‐order symmetry leads to balanced flow structures and vanishing torque. Near *α* ≈ 55° (−5°), the rotational speed reaches a second maximum but in the opposite direction, because the stacking chirality is reversed. More details are provided in real‐time in Movie S2.

For all configurations considered in this section, the combined structure always remains centrally symmetric. Under such symmetry, the resultant aerodynamic force passes through the centre of mass and therefore does not induce eccentric translational motion. Instead, the aerodynamic torque generated by asymmetric force distribution about the centre drives purely rotational motion. When the structural symmetry is perfectly preserved, both pressure and shear stress distributions remain balanced, resulting in zero net torque and a stationary state. Once a small angular misalignment is introduced, the symmetry of the flow field is broken, leading to an imbalance in aerodynamic forces on the two paperflakes. This imbalance generates a net torque about the centre of mass, initiating rotational motion. The rotation is sustained until viscous effects balance the driving torque, leading to a steady‐state spinning condition.

### 2.4 Cluster‐Induced Orbiting

**FIGURE 4:**
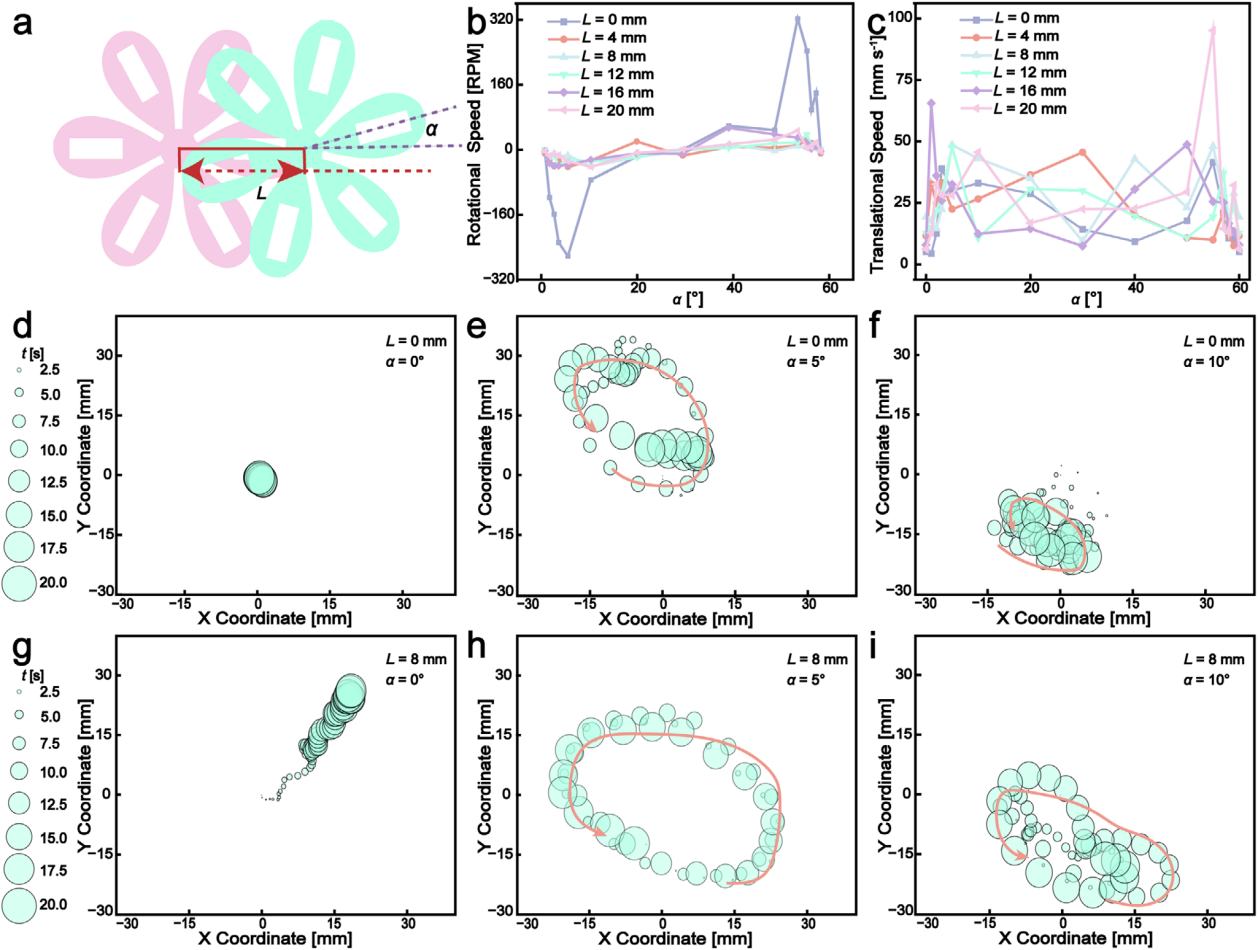
Orbital motion of paired paperflakes. (a) Schematic of a paperflake pair parameterized by relative angle *α *and separation distance *L*. (b) The measured rotational speed as a function of *α *and *L*. (c) Translational speeds of the paperflake pair at different *α *and *L*. Each data point represents the mean behavior over multiple rotational cycles within a 60 s observation. (d–f) Motion trajectories of paperflake pairs with *L* = 0 mm at *α  *= 0°, 5°, and 10°. (g–i) Motion trajectories with *L* = 8 mm at *α  *= 0°, 5°, and 10°.

Beyond the *α *(relative angle), the *L* (separation distance) also governs the aerodynamics of paired paperflake clusters (Figure 4a). Here, we systematically varied *L*, from 0 to 20 mm with 4 mm increment, and measured the corresponding rotational and orbital dynamics. For all non‐zero *L* values, the spinning behavior are insensitive to *α* (Figure 4b). The spinning rate mostly ranges between ± 40 rpm, however observed in a random manner.

To quantify the orbital behavior, we recorded mean translational speeds at different *α* and *L* over a 60s stable gliding. Results shown in Figure 4c indicates that *L* strongly influences the orbital behavior of paperflake pairs, yet no simple patterns were found. At a fixed *α*, increasing the *L* does not result in a monotonic increase of the travelling distance. We also calculated the effective motion radius of the paperflake pair, which provides an intuitive measure of the spatial extent of their motion (Figure S6). Though in Figure 4b,c no monotonic dependence of either rotational speed or orbital displacement on the *L* was observed across the investigated parameter range. Instead, the scattered distribution reflects the sensitivity of the system to small geometric variations in aerodynamic behaviors.

To better visualize for the orbiting motion, we recorded trajectory maps of the paperflake pair over 20 s of flight (Figure 4d–i, Figure S7). Figure 4d–f presents the trajectories for *α* = 0°, 5°, 10°, at *L* = 0 mm. Only the strictly overlapped cluster (*α  *= 0°, *L* = 0 mm) stays still in mid‐air (Figure 4d). An increase in *α* brings in the orbital motion (Figure 4e,f). Figure 4g–i presents trajectories for *α* = 0°, 5°, 10°, at *L* = 8 mm. Since introducing 𝐿 alone breaks central symmetry but preserves bilateral symmetry, the pair exhibits a directional gliding at *α* = 0°. Other clusters display relatively large orbiting trajectory upon increasing the *α*. More details are provided in real‐time in Movie S3. Notably, *L* redistributes aerodynamic forces across the clustered structure where central symmetry and mirror symmetry both vanish where *α ≠* 0, *L ≠* 0. The structural asymmetry introduced by 𝛼 no longer translates aerodynamic forces entirely into spinning motion. Instead, part of the aerodynamic load is redistributed into orbital motion. As a result, under the same angular offset, the rotational speed at 𝐿 *≠* 0 can be two or three times lower than that observed at zero distance. A consistent observation is that higher spinning velocities correspond to more confined orbital range, i.e. shorter centroid trajectories. This indicates that rotational and orbital motion are coupled modes of aerodynamic response to structural asymmetry. To illustrate this mechanism, Figure S8 shows the force decomposition and torque generation. The resultant aerodynamic force is resolved into drag *F_d_* (along the inflow) and lift *F_l_* (perpendicular). The drag balances gravity to maintain suspension. Due to geometric asymmetry, the aerodynamic centre is offset from the centre of mass, thus the lift together with wall shear stresses τ generates a net torque that drives rotation. As rotation increases, viscous shear opposes the motion, reducing the torque until a steady state is reached. Besides, it is important to note that total displacement alone does not fully capture the orbital dynamics, as the clusters can transition between multiple orbital states. In such cases, a portion of the measured displacement arises from translational shifts between distinct orbital configurations, rather than from continuous rotation itself.

### 2.5 Heat‐Induced Tuning of Cluster Spinning

**FIGURE 5:**
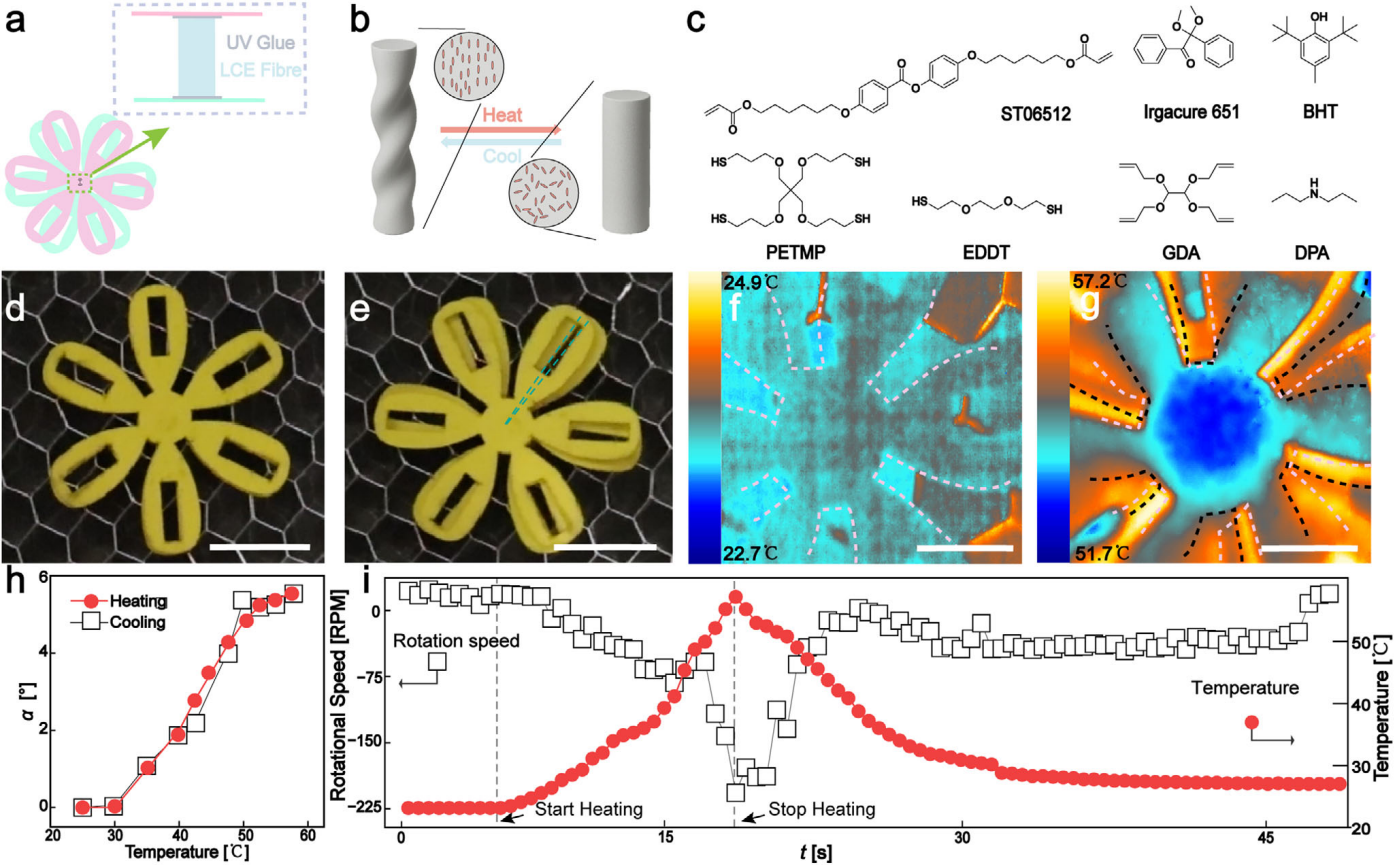
Heat‐flow‐modulation of mid‐air spin. (a) The design of heat‐controlled spinner. The structure is composed of two vertically stacking paperflakes integrated by an LCE fiber actuator at the centroids. (b) Schematic illustration of torsional deformation in fiber actuator induced by the thermal responsiveness of the LCE material. (c) Chemical structures of the molecules used in the LCE. (d, e) Photographs of the paired paperflakes inside the wind tunnel before (d) and after (e) flow heating. Scale bars are 10 mm. (f–g) Infrared thermal images of the paired paperflakes before (f) and after(g) flow heating. Scale bars are 5 mm. (h) Change of *α* as a function of temperature. (i) Recording of rotational speed upon applying and cessation of flow heating.

Building on the geometric tuning of cluster aerodynamics, we have shown that the *α* plays a decisive role in governing the spinning rate, with the peak observed around 5°. To move beyond static function, a twisted actuator was integrated between two paperflakes at *L* = 0 mm to achieve a flyer with mid‐air tunable performance. The actuator is made of a twisted LCE fiber that dynamically adjusts *α* upon reversible heat‐induced deformation. Details of the design are described in Figure 5a.

The key design principle is to achieve a highly sensitive angular response to environmental stimuli (Figure 5b). Heat flow was selected as the triggering signal, delivered by a heat gun to pre‐heat the air before entering the wind tunnel. For this purpose, we adopted an LCE capable of efficient torsional actuation under mild thermal inputs [34, 35]. The monomer mixture (Figure 5c) for LCE synthesis was first injected into a silicone tube and polymerized via a thiol–Michael addition reaction to form a prepolymer. The prepolymer was then removed and mechanically twisted, after which a UV‐induced thiol–ene reaction was employed to permanently fix the twisted configuration. This process yields a 0.56 mm diameter twisted actuator that produces reproducible torsional deformation under heating. The steps of LCE preparation are shown in Figure S9. Figure S10 presents the experimental setup for measuring the torsional response of a LCE fiber and the time‐dependent rotation angle during heating from 23°C to 70°C and subsequent cooling, confirming the material's reliable and reversible actuation. The tangential force measurement is shown in Figure S11.

An LCE fiber with a length of 1.5 mm was sandwiched between two paperflakes using UV‐curable adhesive (Figure S12). Upon heating from room temperature to around 55°C through the heat flow, the LCE sandwiched paperflake pair reconfigures by increasing the *α* up to 7° (Figure 5d,e). Thermal camera images shown in Figure 5f,g indicate the heating distribution around the central areas of the flyer upon weak and strong heat flows that presented in Figure 5d,e. Figure 5h plots the *α* of the paperflake pair as a function of temperature, demonstrating a reversible change of *α* in a cycle, indicating an opportunity for the mid‐air tuning of the spinning rate.

Finally, we performed a proof‐of‐principle demonstration to show that introducing a heat flow into the wind tunnel environment can reversibly modulate the spinning rate. A schematic illustration of the heated airflow supply system is shown in Figure S13. The spinning rate evolution is presented in Figure 5i. Prior to heating, the flyer remained at 0 rpm inside the wind with random drift. Upon flow heating, the spinning rate gradually increases to 220 rpm. After ceasing the heat, the spinning rate slowly returns to the original condition, indicating a reversible mid‐air switching of the spinning.

### 2.6 Collective Clustering of Multiple Paperflakes

**FIGURE 6:**
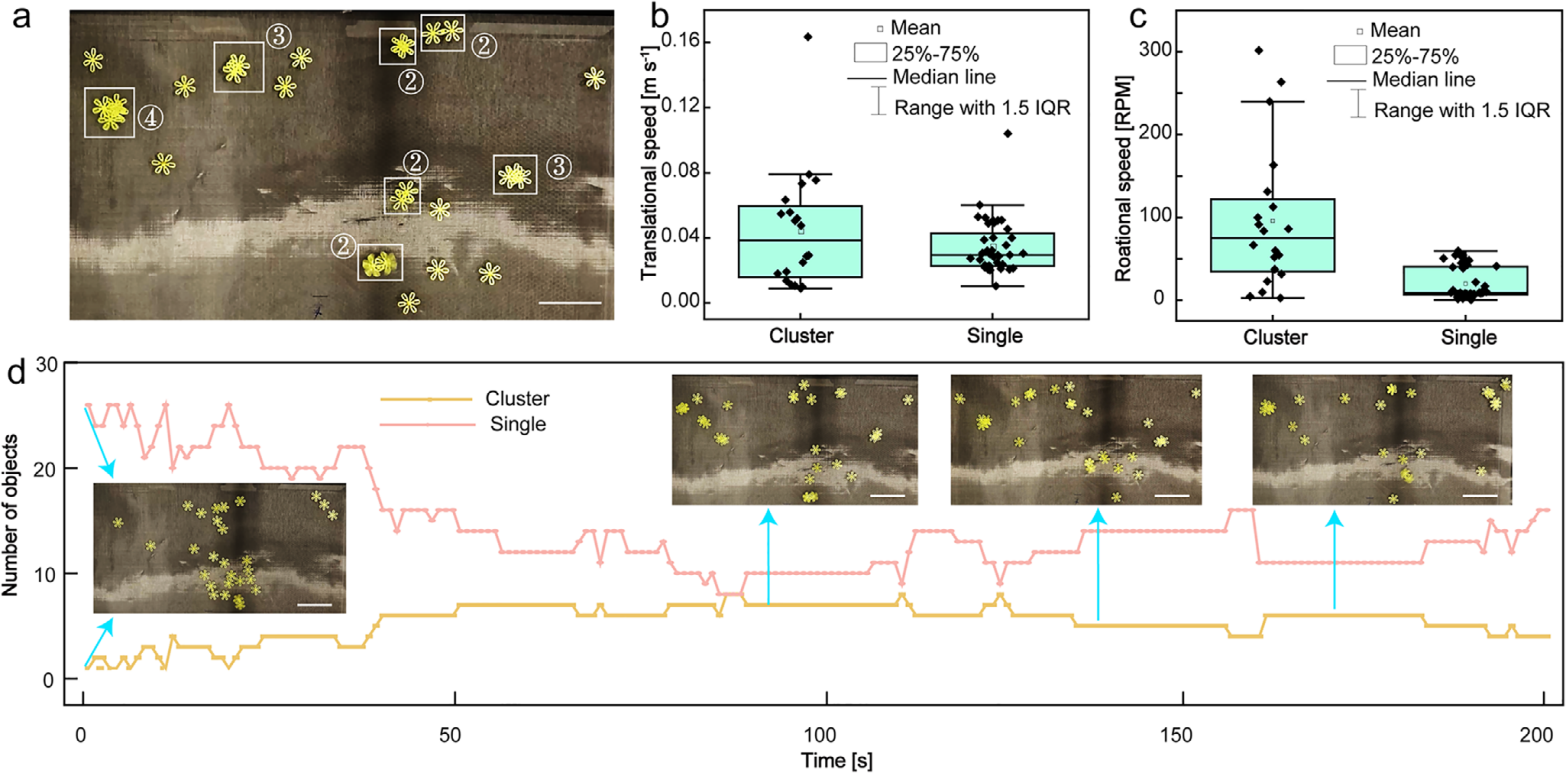
Collective dynamics. (a) Representative video snapshot with clustered configurations highlighted by white boxes. The numbers denote the number of paperflakes in each cluster. (b, c) Translational velocities and rotational velocities of single paperflakes and clusters (lasting longer than 3 s), sampled at different spatial locations and time points within the wind tunnel. (d) Temporal evolution of the numbers of single paperflakes and clusters during the self‐assembly process, with representative snapshots at selected time points. Scale bars: 10 cm.

Individual flyers spontaneously connect with one another through vortex interactions. To explore the clustering dynamics, we performed experiments by simultaneously releasing 28 individual paperflakes into the wind tunnel. Upon entering the airflow, the paperflakes undergo dynamic self‐assembly accompanied by cluster formation, breakup, spinning, and orbital motions. Representative results are summarized in Figure 6, and the full process is visualized in Movie S1.

Figure 6a shows a representative snapshot in which several clustered configurations are identified. Cluster sizes ranging from two to four paperflakes are observed, with two‐flake clusters being the most prevalent configuration throughout the experiment. Figure 6b,c compares the translational and rotational velocities of isolated and clustered paperflakes. While isolated paperflakes exhibit narrower velocity distributions, clusters display broader ones. Notably, the minimum translational velocities of single and clustered paperflakes are comparable. However, the maximum translational velocity of clusters reaches approximately 1.7 times that of isolated individuals. An even stronger contrast is observed in the rotational dynamics, where clustered configurations achieve rotational speeds up to nearly five times those of isolated paperflakes.

Figure 6d presents the temporal evolution of the numbers of isolated paperflakes and clusters during the selfassembly process. The system alternates between phases of rapid aggregation and relative stability, during which the populations of clusters and single paperflakes remain approximately constant for extended periods. Representative snapshots at such characteristic time points are included to illustrate the corresponding spatial configurations. During the dynamic assembly process, clustered configurations generally tend to exhibit enhanced translational and rotational motions compared with isolated paperflakes. However, in some particular cases, some larger clusters containing more than three paperflakes display relatively stationary after formation. These observations highlight the complexity of manybody aerodynamic interactions and the decisive role of flyer's geometry in aerodynamic behavior.

Figure 6d presents the temporal evolution of the numbers of isolated paperflakes and clusters during the self‐assembly process. The system alternates between phases of rapid aggregation and relative stability, during which the populations of clusters and isolated paperflakes remain approximately constant for extended periods. Representative snapshots at these characteristic time points are included to illustrate the corresponding spatial configurations. Throughout the dynamic assembly process, clustered configurations generally exhibit enhanced translational and rotational motions compared with isolated paperflakes. However, in certain cases, larger clusters containing more than three paperflakes display relatively stationary behavior following their formation. These observations highlight the complexity of many‐body aerodynamic interactions and the decisive role of cluster geometry in governing aerodynamic behavior.

## 3 Discussion

We note that while the paperflake is geometrically inspired by natural snowflakes, the two systems operate in different aerodynamic regimes. Natural snowflakes, with characteristic sizes of 1–3 mm and falling velocities of 20–100 cm/s, correspond to Reynolds numbers (*Re*) on the order of 10–100. The paperflake models, with a diameter of 29 mm and wind tunnel flow velocities of 15–200 cm/s, operate at *Re* ∼ 300–2000. The snowflake therefore serves as a geometric metaphor rather than a dynamic analog in this study. Two points regarding the role of Reynolds number are worth noting. First, the process by which individual flyers form clusters is driven by aerodynamic wake interactions, which naturally differ across Re regimes. However, our study focuses on the post‐formation aerodynamics, specifically, how the asymmetric geometry governs aerodynamic behavior after clustering has occurred. Second, the central physical mechanism, that is the cluster‐induced geometric asymmetry drives spinning, is a general principle that operates across a wide range of *Re*. To support this, we conducted additional experiments using smaller paperflakes (diameter ≈ 6 mm) at a lower airflow velocity of 0.16 m s^−^^1^, yielding *Re*. ≈ 60, which falls within the typical range of natural snowflakes. In these low‐Re experiments, isolated paperflakes remained stably suspended without rotation, whereas clustered paperflakes clearly exhibited spinning and orbital motions, as shown in Movie S4.

By overlapping the centroids of the paperflake pair (*L* = 0), our experiments tested the influence of different *α*  on the spinning velocity in Figure 3. The angular velocity exhibited the most enhancement upon a 5° change in relative angle. This significant change of spinning highlights a high sensitivity in aerodynamic behavior and a strongly dynamical response under minimally induced asymmetry. We believe certain nonlinear characteristics of aerodynamics are engaged in the spinning motion. The clustered structure likely triggers a symmetry‐breaking bifurcation, driving the state to change from a stable stillness into a stable spinning. The newly established equilibrium is governed by deterministic limit cycle attractor rather than a chaotic regime that usually found in vortex shedding driven random fluctuation. Within this nonlinear regime, the stable spinning motion inherently absorbs perturbations and demonstrates self‐stabilization [36].

We further show that the geometric parameters *α* and *L* govern distinct dynamical modes: *α* primarily controls spinning, while *L* introduces longer distance gliding that redistributes aerodynamic load from spinning to orbital motion. These findings suggest potential opportunities for future research in flying micro robots. Specifically, to achieve mid‐air steering, one may apply the fine‐tune of the relative angle at larger separation distance between two paperflakes, in order to achieve sophisticated control of two‐dimensional trajectories. The coupling between spinning and orbiting motion also enriches the design space for aerodynamic manipulation using simple clustered geometries.

We note that the flow conditioning in the wind tunnel further minimises external disturbances. The airflow passes sequentially through a honeycomb structure followed by a fine metal mesh (thickness: 0.1–0.2 mm, pore size ≈ 0.1 mm) prior to entering the test section. While the honeycomb may introduce cell‐scale velocity variations, the downstream mesh layer acts as a secondary flow straightener that effectively suppresses these residual non‐uniformities. Given that the characteristic size of the paperflake (∼29 mm) is orders of magnitude larger than both the honeycomb cell size and the mesh pore size, the flyers experience a spatially averaged flow field. Consistently, experimental observations show that a single paperflake and paperflake pairs with α = 0° remain stably suspended with minimal rotation under the same conditions, indicating that any residual flow non‐uniformities are insufficient to induce aerodynamic asymmetry. This supports the conclusion that the observed spinning and orbital motions are governed primarily by cluster‐induced geometric asymmetry rather than externally imposed flow perturbations.

To achieve a fast responsive, energy efficient mid‐air flyer steering necessities stimulus‐deformable actuators quick responsiveness. The current LCE fiber demonstrates proof‐of‐principle tunability, however, the heat flow stimulation method and flyer's thermal capacity limit the responding speed. Promising alternatives include the uses of sunlight driven LCE actuator [37, 38], humidity‐sensitive bilayer actuators [39], and lightweight materials capable of faster, programmable deformations [9].

The cluster‐induced spinning mechanism demonstrated here is a general principle widely observed across wind‐assisted flyers in nature, from snowflake clusters and aggregated spores to paired dandelion seeds, suggesting that the design rules derived from this model system are broadly applicable beyond the specific paperflake geometry. The approach offers natural scaling possibilities: the single flyer design can be miniaturized toward micro‐scale aerial systems, while clustered configurations of larger flyers open pathways toward up‐scaled passive aerial platforms with enhanced aerodynamic performance. Regarding applications, we would like to note that, incorporating stimuli‐responsive color‐changing films into the flyer structure would enable visual environmental sensing, where changes in ambient conditions such as temperature, humidity, or chemical exposure are directly encoded as color signals readable from a distance [8, 14, 20, 40, 41, 42, 43]. Up‐scaled clusters of flyers could be designed to collectively carry small payloads, leveraging the enhanced gliding trajectories and prolonged airborne time demonstrated in this work.

In conclusion, this study presents a physical model of a wind‐assisted flyer exhibiting cluster‐dependent aerodynamic behavior. The flyer, termed a paperflake, is laser‐cut from standard office paper and features sixfold central symmetry with lobes containing hollow regions that introduce porosity. A single paperflake can remain suspended in a steady wind flow with minimal lateral drift (<1/4 body length) and limited rotational deviation (approximately 10°) during a 1‐min mid‐air flight. CFD simulations reveal a centrally symmetric wind field and a significant contribution from the downstream low‐pressure zone, both of which underpin the flyer's airborne stability. Experimentally, we characterize the effect of the clustering parameters – i.e., the separation distance and relative angle when two paperflakes are joined – on their aerodynamic behavior. The spinning velocity peaks at relative angles of 10° and 55° (−5°), while the orbital trajectories show random variations but are generally enhanced by larger separation distances and lowering spinning rates. CFD analyses indicate a noticeable pressure asymmetry even with 1° misalignment between two flyers, which accounts for the observed cluster rotation. To enable wireless, in‐flight control of the spinning rate, we integrated a twisted LCE fiber actuator between two flyers. This actuator is heat‐responsive and can reversibly adjust the relative angle from 0° to approximately 6°. By introducing a controlled heat flow into the wind tunnel, mid‐air tuning of the spinning rate was achieved, allowing a switch between 0 and 220 rpm rotation. This study reveals how geometric asymmetry can induce self‐spinning and orbital motion in clustered objects suspended in air. Technically, it demonstrates the potential to actively modulate mid‐air microrobotic function through the integration of responsive materials.

## 4 Experimental Section

### 4.1 Materials in Brief

ST06512 (4‐(6‐(Acryloyloxy)hexyloxy)phenyl 4‐(6‐(acryloyloxy)hexyloxy)benzoate) was obtained from SYNTHON Chemicals. GDA (tetraallyloxyethane) and DPA (dipropylamine) were obtained from TCI. EDDT (2,2′‐(ethylenedioxy)diethanethiol), PETMP (pentaerythritol tetrakis(3‐mercaptopropionate)), BHT (2,6‐di‐tert‐butyl‐4‐methylphenol), Irgacure 651 (2,2‐dimethoxy‐2‐phenylacetophenone), and Disperse Red 1 were obtained from Sigma‐Aldrich. All chemicals were used as received.

### 4.2 Paperflake Fabrication

Paperflakes were fabricated by laser cutting from colored office paper. The paper used was commercially available colored copy/printing paper. The 2D paperflake model and specific cutting parameters (including laser wavelength, material type, power, etc.) are provided in Figure S3.

### 4.3 LCE Fabrication

The reaction mixture, containing 0.27 mmol ST06512, 0.12 mmol EDDT, 0.03 mmol PETMP, 0.015 mmol GDA, 0.5 wt.% DPA, 1.5 wt.% Irgacure 651, and 1 wt.% BHT, was thoroughly combined at 80°C to form a homogeneous solution, which was injected into a PDMS tube and maintained at 80°C for 24 h to generate the prepolymer through a thiol–Michael addition reaction. Subsequently, the prepolymer was removed from the PDMS tube, mechanically stretched and twisted, and polymerized with UV source of 365 nm, 180 mW cm^−^^2^, 5 min, to yield the fiber actuator.

### 4.4 CFD Simulation

Numerical simulations were carried out using ANSYS Fluent 2023R2, with all parameters detailed in the Numerical Simulation Details section in the Supporting Information. The inlet was specified as 0 Pa gauge pressure, all pressures in the simulation are reported relative to this reference value.

### 4.5 Wind Tunnel Setting

The wind tunnel has a cross‐sectional size of 420 mm × 420 mm and is constructed from an aluminium alloy frame with acrylic glass panels. It consists of three main sections: a driving layer, composed of fans and 3D‐printed inlet and outlet ducts; a stabilizing layer, made of a 30 mm‐thick aluminum honeycomb panel and a stainless‐steel mesh; and an experimental layer. For the paired paperflake experiments, the airflow velocity was approximately 0.30 m s^−1^ with no changes. The wind speed was measured at the same height as the samples using an AOPUTTRIVER AP‐856A handheld anemometer. Each measurement was repeated five times, and the average value was used for analysis. The wind tunnel experiments were conducted with flyers suspended above a honeycomb mesh, which intentionally confines their motion to a quasi‐2D plane. Importantly, this confinement does not involve mechanical contact between the flyers and the mesh, as confirmed by the side‐view observations in Movie S5. We notice that this represents a constrained rather than free‐flight condition. However, it serves as a necessary experimental simplification that enables systematic study of clustering behavior in a controlled 2D configuration. The wind speed can be tunned between 0.1 and 1.7 m s^−1^ in the wind tunnel.

To induce heated flow a heat gun is used. The temperature at the outlet of the heat gun is approximately 85°C, and it takes approximately 18 s of continuous heating for the airflow inside the wind tunnel to reach its maximum steady‐state temperature.

### 4.6 Data Analysis

Rotational and orbital velocities were measured using a camera operating at 120 fps and analyzed with Tracker software. The rotational speed was obtained from the instantaneous angular velocity extracted at each frame and subsequently averaged over a defined steady‐state time window after the flyer cluster had stabilised in the airflow. In Sections 2.3 and 2.4, the averaging was performed over a 10 s interval, whereas in Section 2.6, a 3 s window was used. The translational speed in Section 2.4 was evaluated over a longer duration of 60 s to capture the overall displacement behavior.

### 4.7 Artificial Intelligence Generated Content

This manuscript used OpenAI ChatGPT‐5.0 solely for grammar editing to improve readability.

## Author Contributions

H.Z. conceived and supervised the project; V.S. designed the flyer. B.Z. performed the experiments with help from R.C., F.A.K., J.Y., and Q.Y.; B.Z. analysed the data and conducted the CFD simulation; F.L. fabricated the LCE actuator; H.Z., B.Z. wrote the manuscript taking inputs from J.Y and R.C. All authors discussed and contributed to the project.

## Conflicts of Interest

The authors declare no conflicts of interest.

## Supporting information




**Supporting File 1**: advs76560‐sup‐0001‐SuppMat.pdf.

**Supporting File 2**: advs76560‐sup‐0002‐MovieS1.mp4.

**Supporting File 3**: advs76560‐sup‐0003‐MovieS2.mp4.

**Supporting File 4**: advs76560‐sup‐0004‐MovieS3.mp4.

**Supporting File 5**: advs76560‐sup‐0005‐MovieS4.mp4.

**Supporting File 6**: advs76560‐sup‐0006‐MovieS5.mp4.

## Data Availability

The data that support the findings of this study are available from the corresponding author upon reasonable request.
