# Supplementary material for: Emergent Spinning and Orbital Motion in Clustered Wind‐Assisted Flyers
Source: Adv Sci (Weinh). 2026 Jul 11;13(55):e76560. doi: 10.1002/advs.76560 (PMC13355929; doi:10.1002/advs.76560)
Supplement: Supplementary file 1 [file ADVS-13-e76560-s002.pdf]

Supporting Information

Emergent Spinning and Orbital Motion in Clustered Wind-Assisted Flyers

Bingnan Zhou, et al.

This PDF file includes:

1. **Supplementary Figures S1-S18.**
2. **Supplementary Notes.**

   Supplementary Note 2.1 Transition SST turbulence model.

   Supplementary Note 2.2 Numerical simulation results of the paperflake.
3. **References.**
4. **Captions for Supplementary Movies S1-5.**

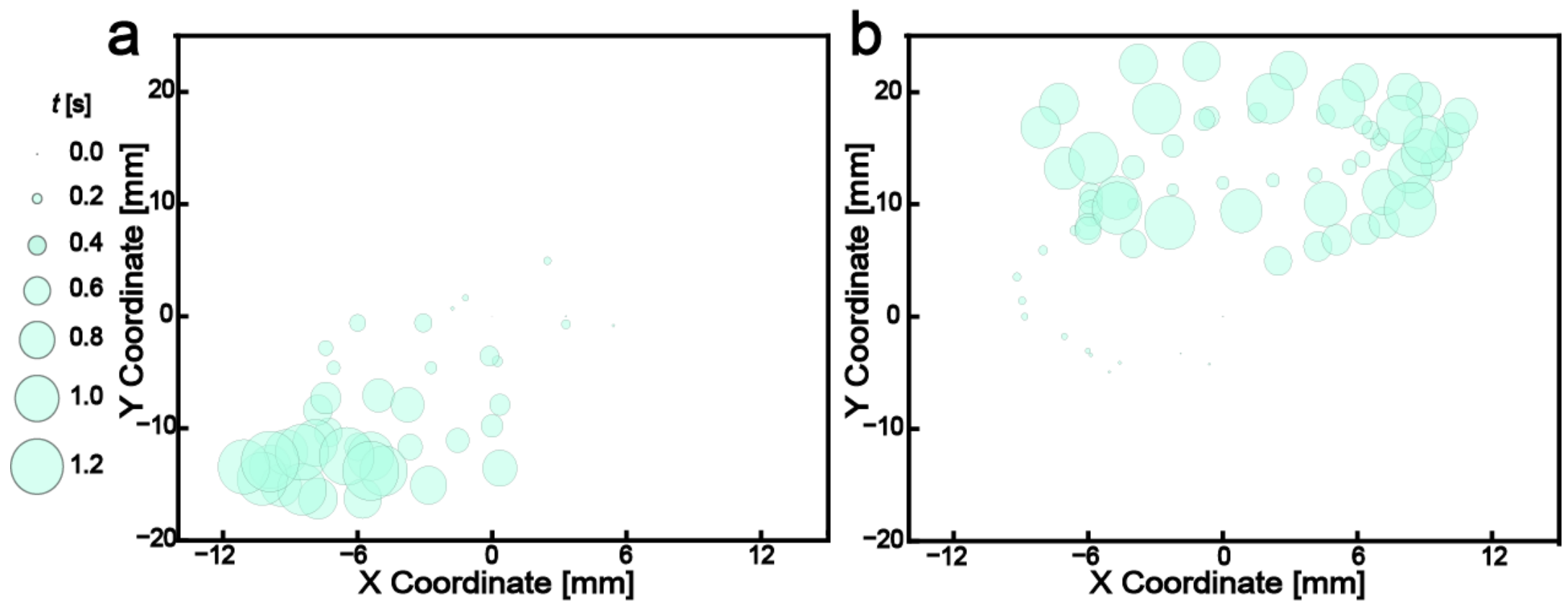

Figure S1. Trajectories of paperflakes during free falling inside the still air. a) Motion trajectory of the centroid of a single paperflake. b) Trajectory of the centroid of a paperflake pair. Both positions are recorded from the top-view angle.

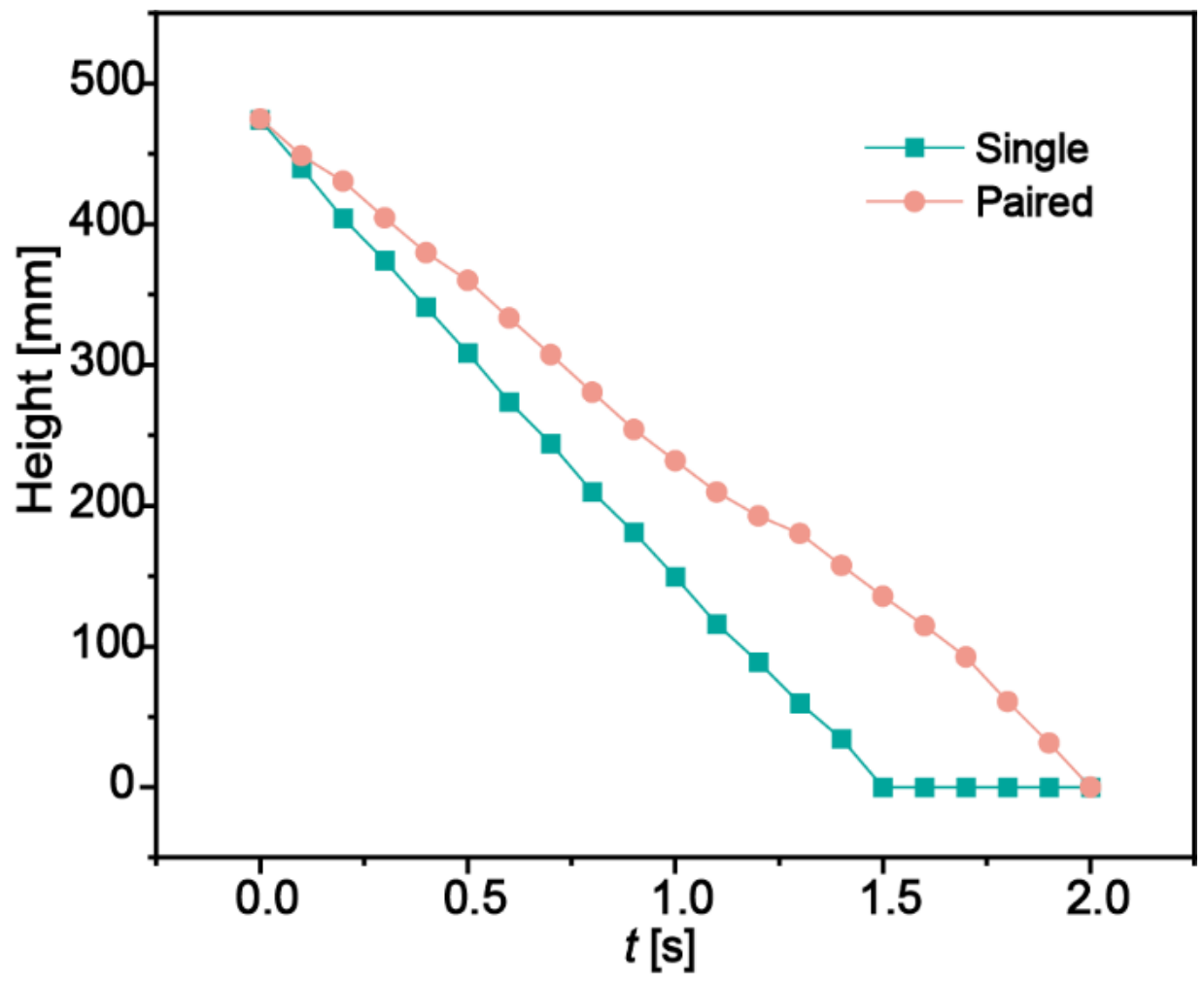

Figure S2. The height changes during the free falling. The centroid of a single paperflake and a paperflake pair are recorded along vertical direction.

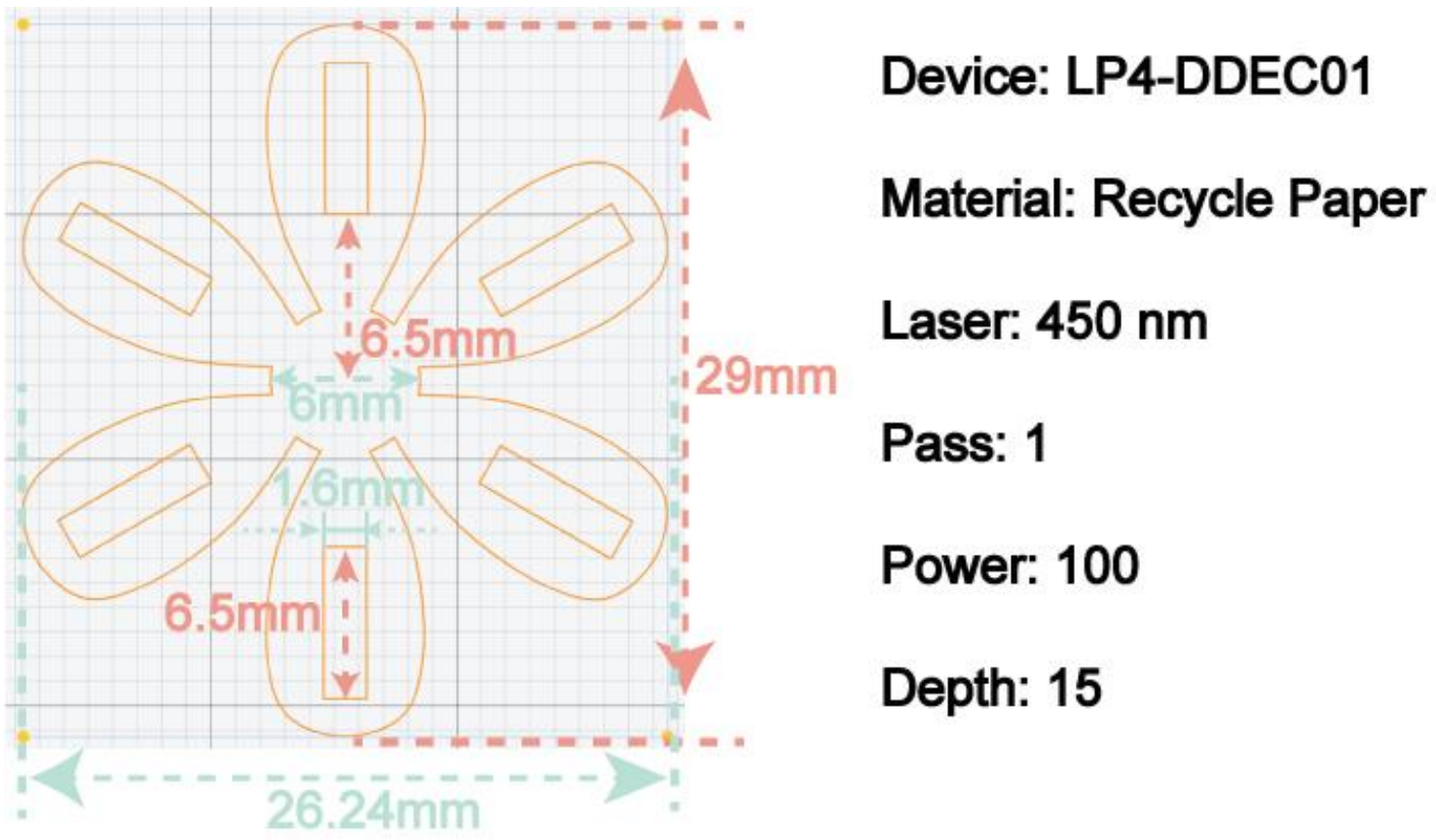

Figure S3. The design of the flyer. The geometrical used for laser cutting, and the list of technical settings.

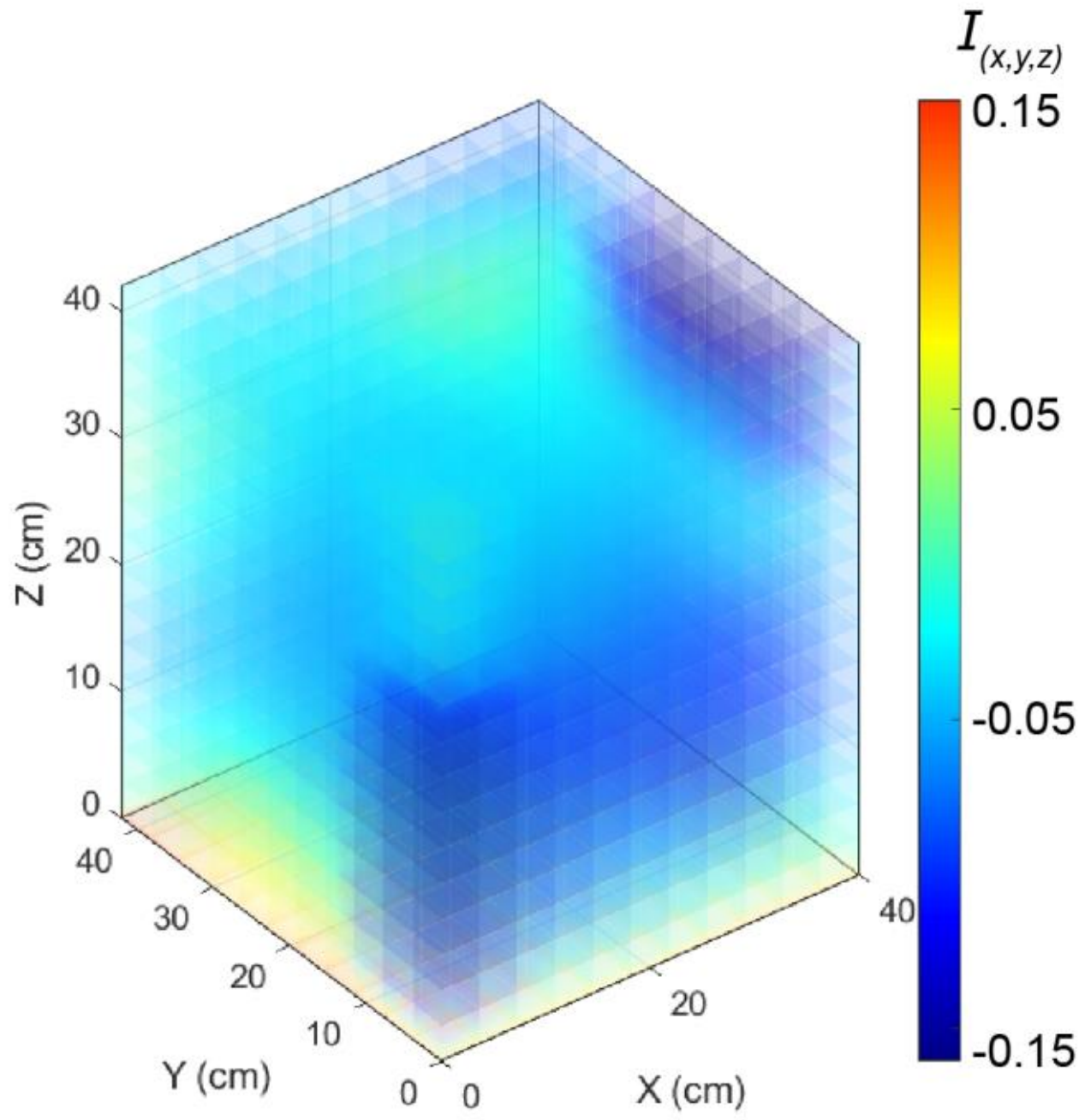

Figure S4. Spatial uniformity of wind flow inside the wind tunnel.

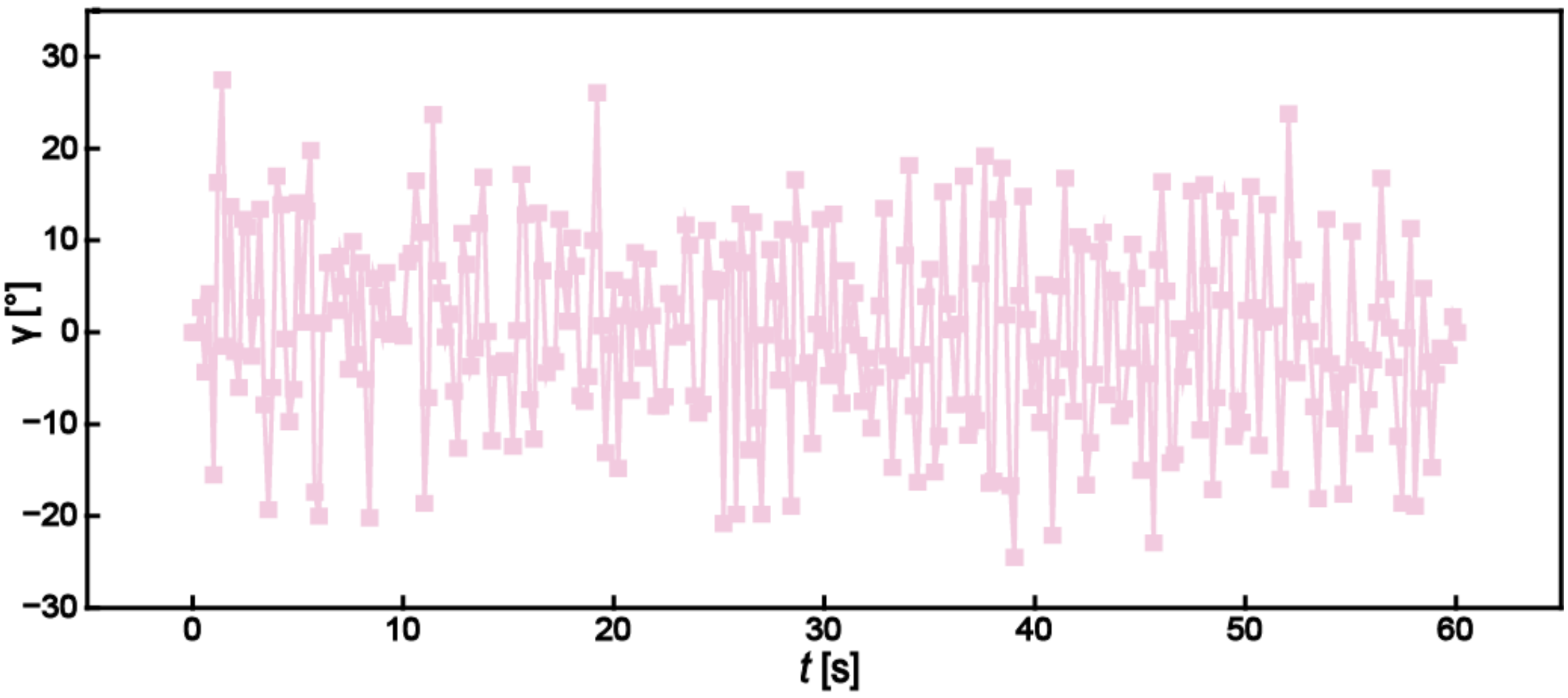

Figure S5. Mid-air stability of a single flyer. The rotational angle γ is recorded to show the time-dependent variation over one minute inside the wind tunnel. The measured data exhibit a mean value of 0.06° and a standard deviation of 10.12°.

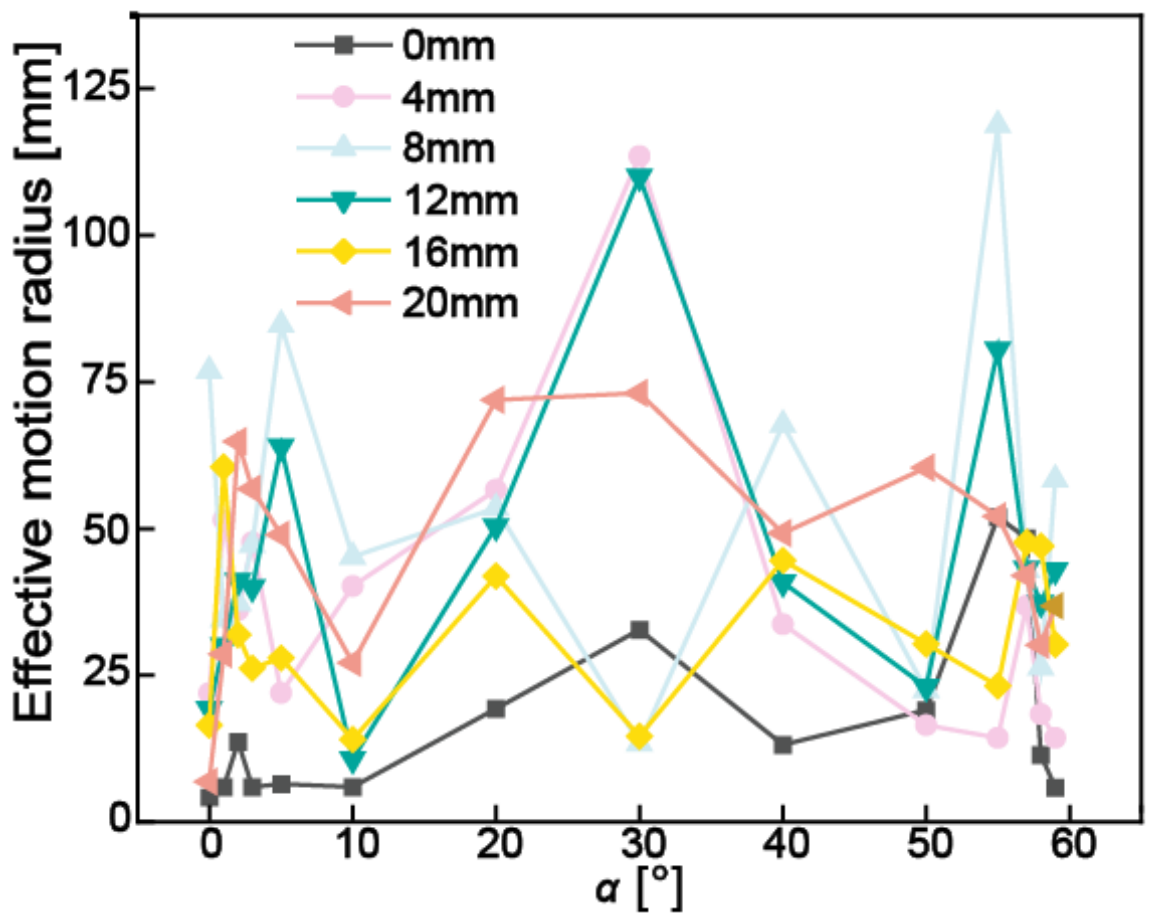

Figure S6. Effective motion radius of paperflake pairs. Experiments were performed using different clustering parameters, i.e., relative angle α from 0 to 60°, and separation distance L from 0 to 20 mm. The effective motion radius was derived from the measured trajectory area (A) projected on the horizontal plane, using approximation, $r = \sqrt{A/\pi}$.

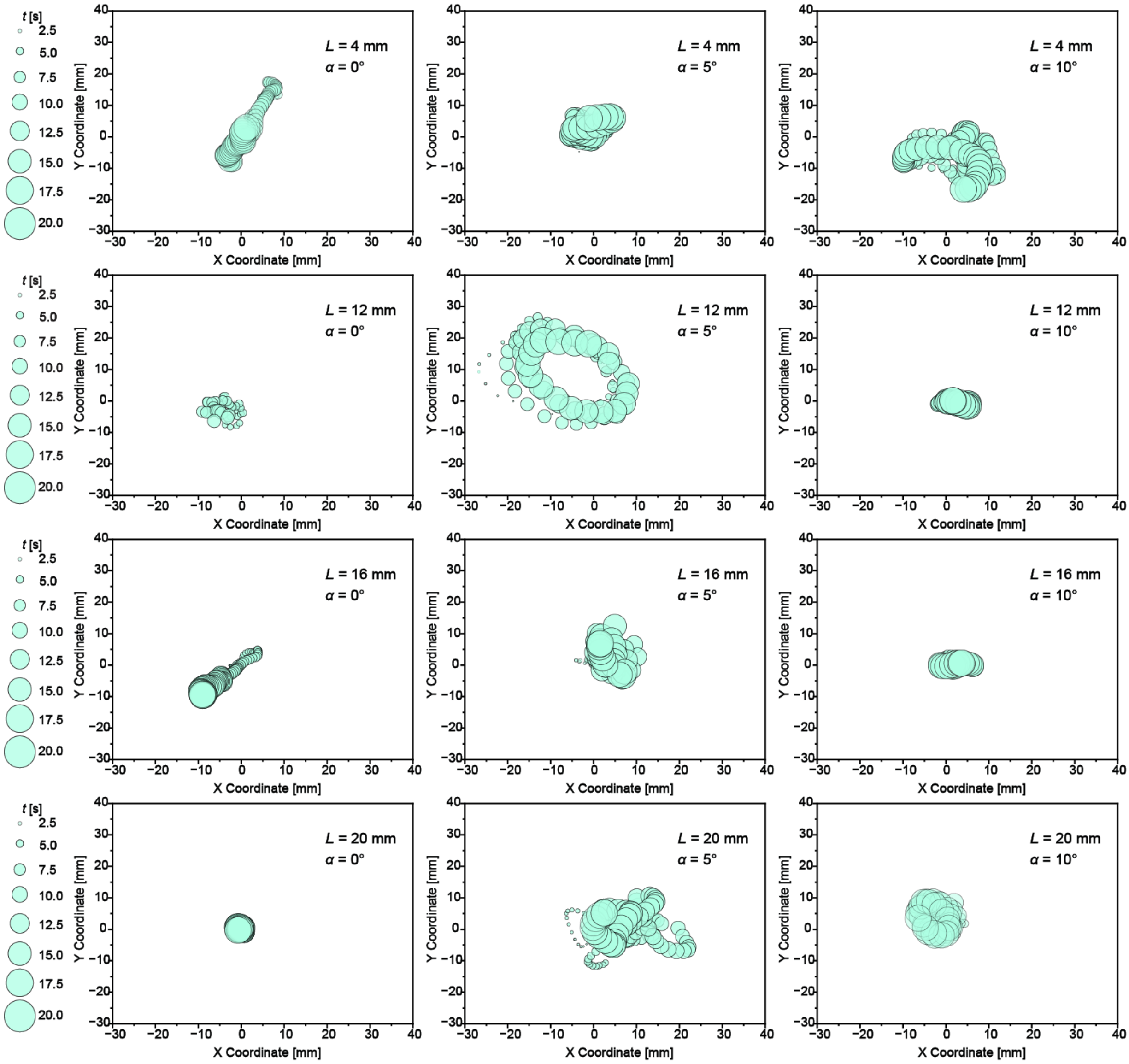

Figure S7. **Measured motion trajectories of paired paperflakes**. The gliding experiments were performed using different clustering parameters, i.e., relative angle α = 0, 5, 10°, and separation distance L = 4, 12, 16, 20 mm.

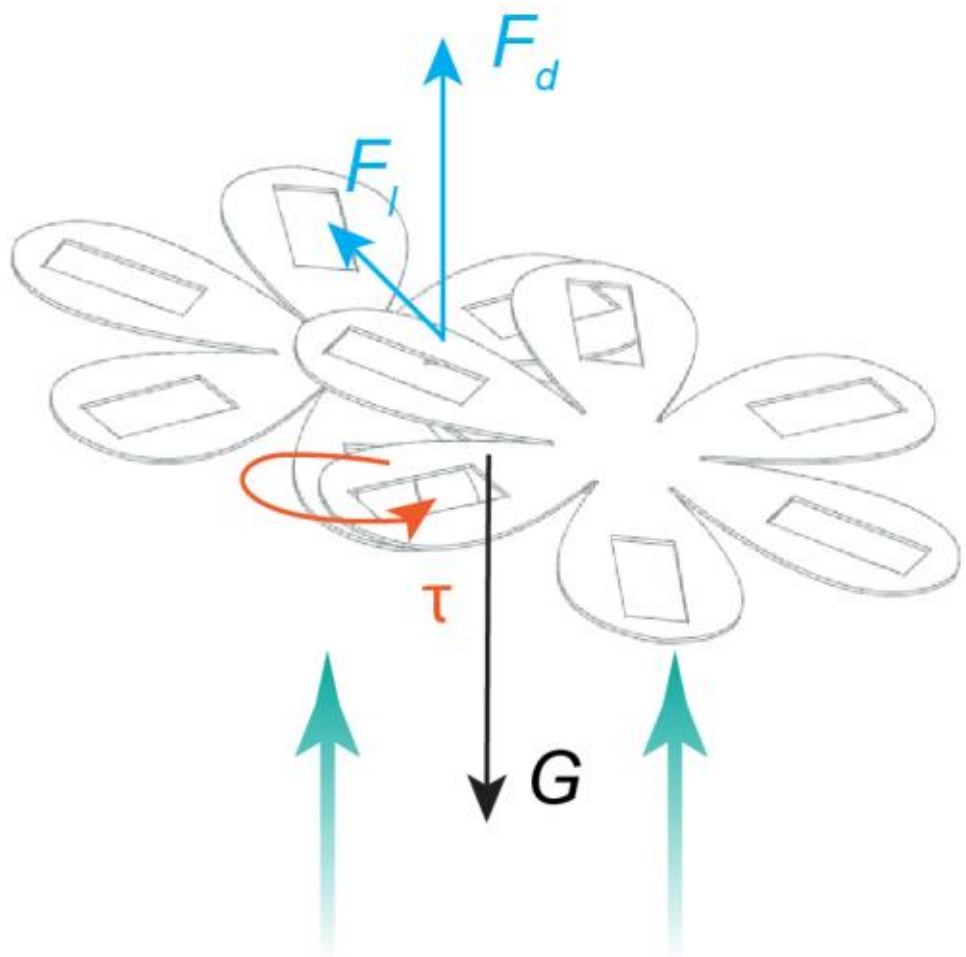

Figure S8. Schematic of force decomposition and torque generation on a paperflake pair.

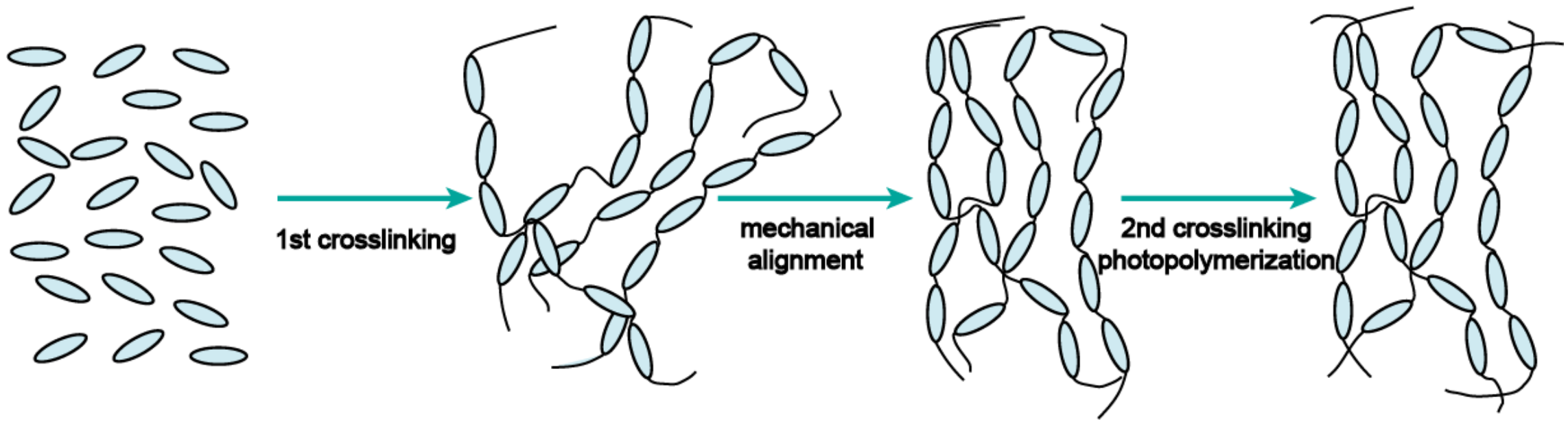

Figure S9. Polymerization steps of LCE preparation. Polymerization steps of LCE preparation. A thiol–Michael addition reaction is taken at the mixture during the first crosslinking to create a loosely crosslinked prepolymer network. The prepolymer is then removed from the silicone tube, mechanically twisted, and subsequently UV-cured in the second polymerization step.

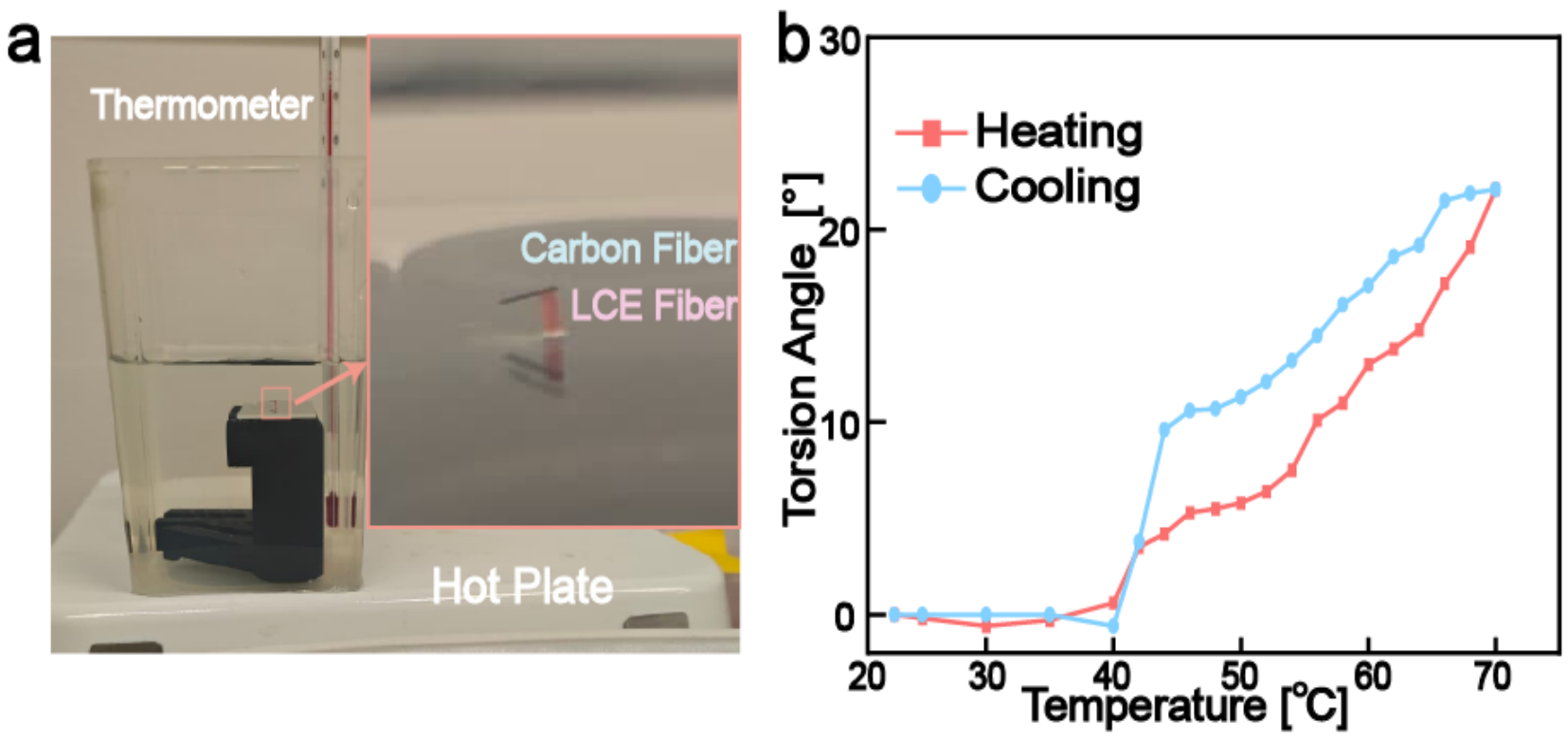

Figure S10. Torsional deformation of the LCE fiber. (a) Water bath setup for measuring the heat induced deformation. The whole actuator is immerged inside the water for homogeneous heating. A thermometer is used to measure the temperature of the bath. (b) The temperature-dependent torsional angle of the fiber during one heating-cooling cycle. The length of LCE fiber is 2 mm.

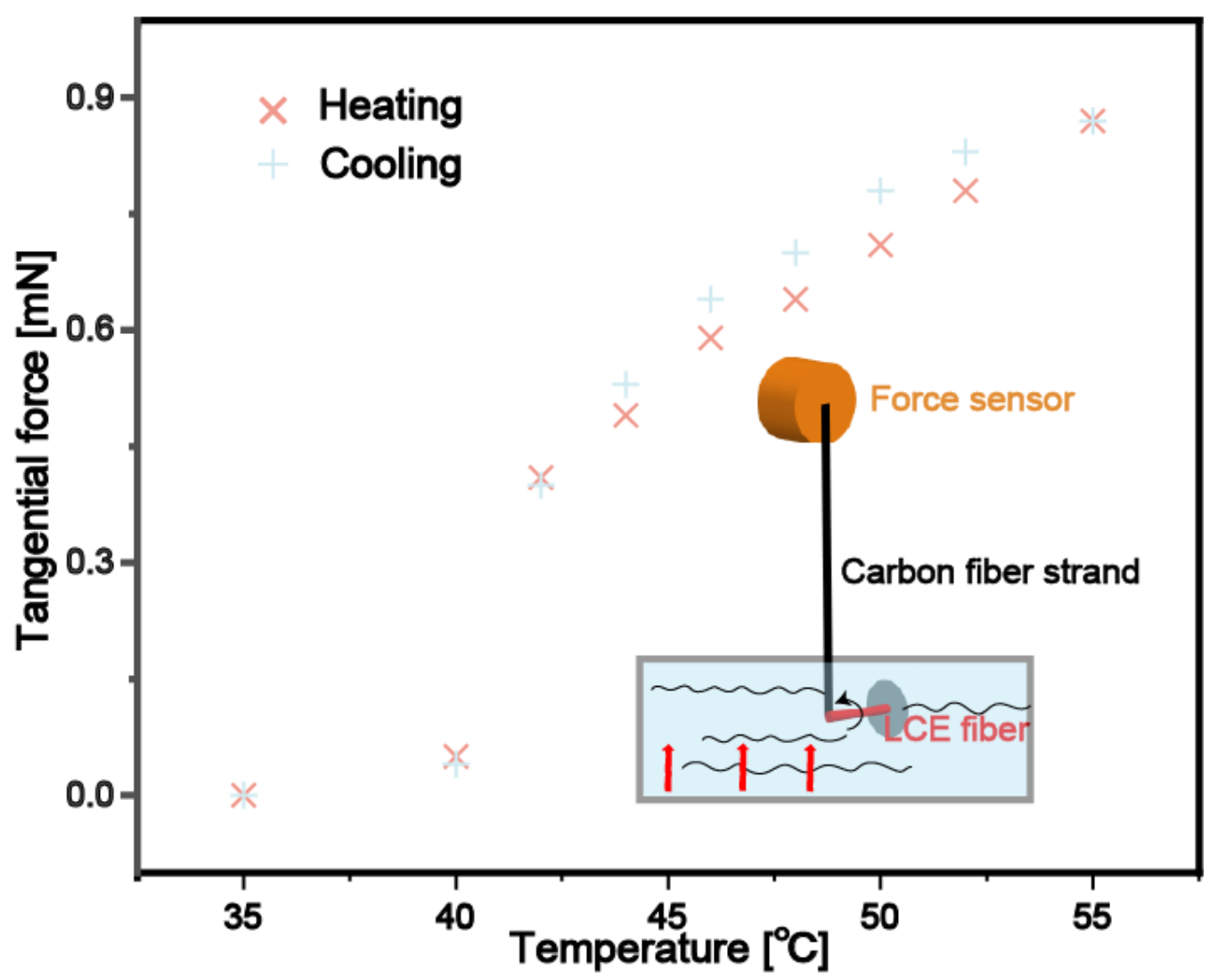

Figure S11. Tangential force measurement. A LCE fiber with a length of 2 mm was heated inside a water bath. The fiber was fixed at one end, and attached at its free end a 20 mm long carbon fiber strand. The free end of the strand pressed against a force sensor, which recorded the tangential force generated by the LCE's torsional deformation upon heating/cooling.

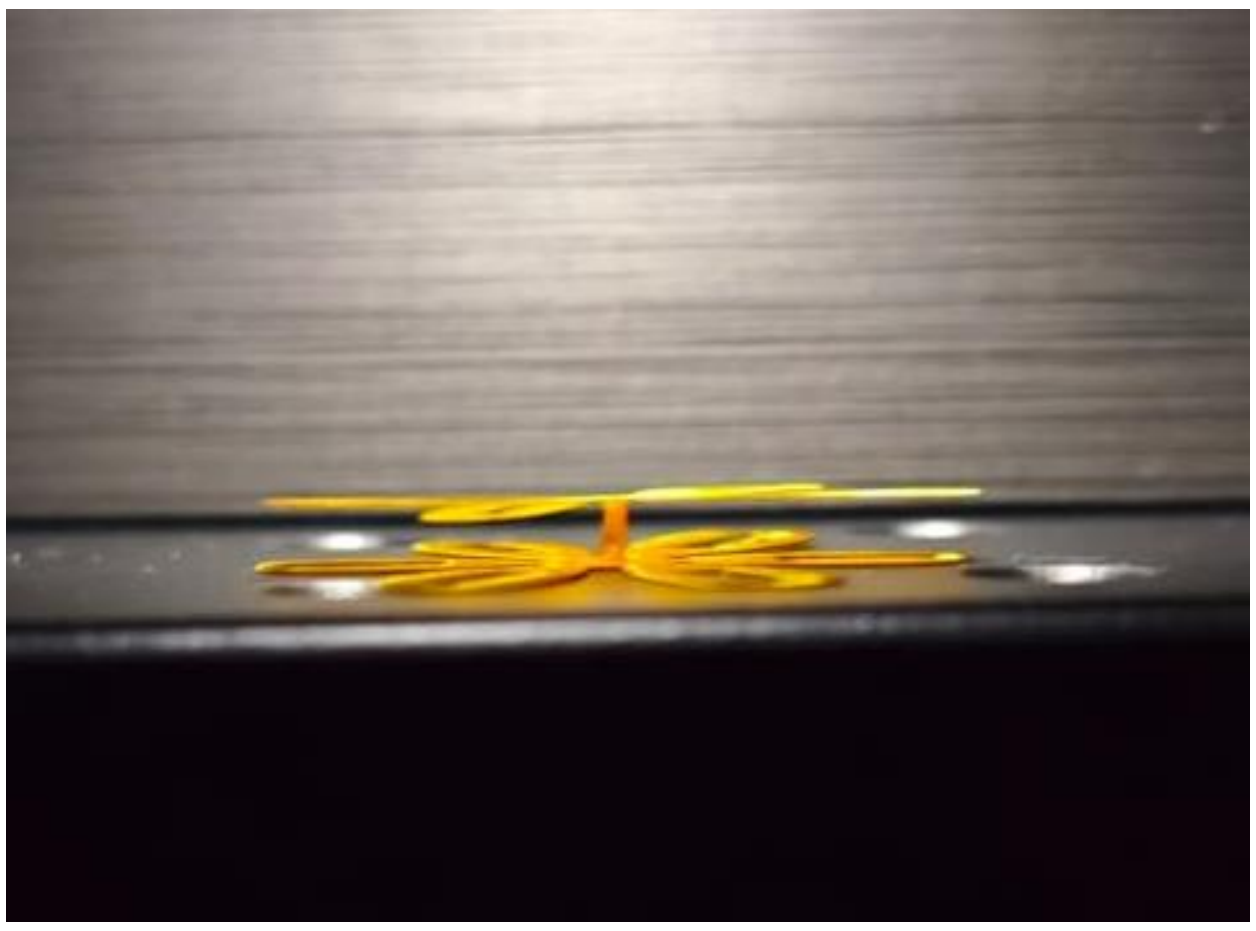

Figure S12. Side-view photograph of the assembled paperflake pair.

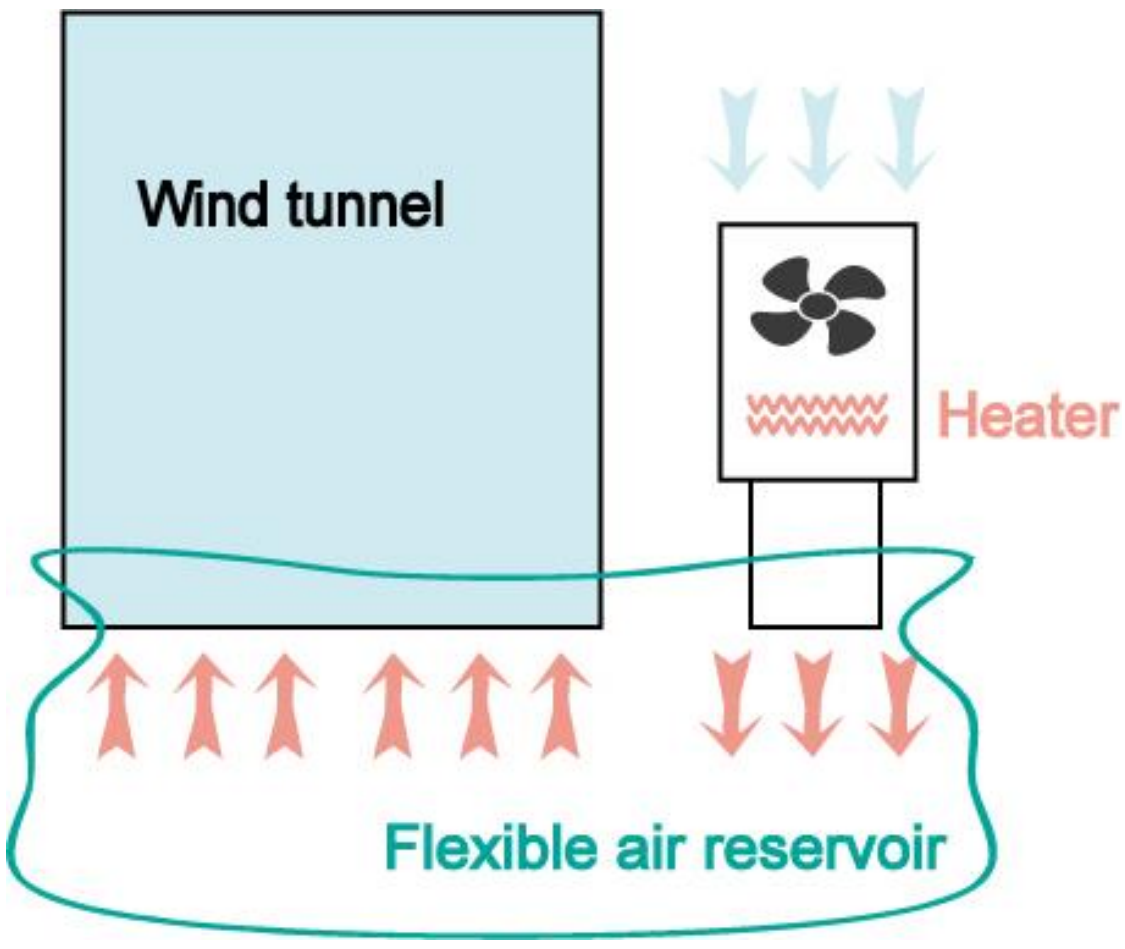

Figure S13. Schematic illustration of the heat-flow stimulation system. The flexible air reservoir is made of a plastic bag with volume about 0.2 m^3, which couple the wind tunnel setup and the heat source. A heat gun is used as the heater to blow heated air into the reservoir with a measured flow rate at the heater outlet about 0.025 m^3 s^{-1}.

2 Supplementary Notes – Numerical simulation details

Numerical simulations were carried out in Ansys Fluent 2023 R2 using the Transition SST turbulence model. A single paperflake has a diameter of 29 mm and a thickness of 0.15 mm, and its geometric shape is shown in Figure S3. The wind-tunnel domain was set to be 180 mm in diameter and 300 mm in height. The fluid material was set as air. Unless otherwise specified, boundary conditions and solver parameters followed software defaults.

The computational mesh was generated using Tet4 elements, with a fluid grid size of 8 mm and a refined paperflake grid size of 0.3 mm, resulting in a total of 365.8k meshes. The simulation was performed by calculating the pressure field distribution, velocity field distribution, shear stress field distributions and torque with the paperflake fixed at the center of the flow field under a constant incoming velocity of 0.3 m s^{-1}.

2.1 Transition SST turbulence model.

The Transition SST turbulence model was employed for CFD simulations due to the transitional Reynolds number regime of the present system. Based on the characteristic diameter of the flyer ($D = 29$ mm) and the inflow velocity used in the simulations (U: 0.3 m s^{-1}), the Reynolds numbers can be estimated as

$$Re = \frac{\rho U D}{\mu} \approx 5.9 \times 10^{2}$$

using the density of air $\rho = 1.2$ kg m^{-3} and dynamic viscosity $\mu = 1.8 \times 10^{-5}$ Pa s. This Reynolds number lies within the transitional regime, where laminar boundary layers, flow separation, wake instability, and localized vortex shedding may coexist. Under such conditions, purely laminar models may not adequately capture the onset of transition and separated flow structures.

The governing equations include the transport equations for turbulent kinetic energy k, specific dissipation rate ω, intermittency γ, and transition onset momentum-thickness Reynolds number $Re_{\theta t}$.[1] Unless otherwise specified, all model coefficients and solver parameters followed the default settings implemented in Ansys Fluent 2023 R2.

The boundary conditions were defined as follows: the inlet was set as a velocity inlet with a constant velocity of 0.3 m s^{-1}, the outlet as a pressure outlet at 0 Pa gauge, and all other surfaces were set as walls with no-slip conditions. Unless otherwise specified, solver parameters followed software defaults.

Figures S14 a-d show iso-surfaces of vorticity magnitude(Q=50) for paperflake pairs at $\alpha = 0°$, 1°, 5°and -5°, $L = 0$. Regarding numerical convergence, the simulations were iterated

until the residual of the continuity equation decreased below 10^{-6}, while all remaining residuals also exhibited stable convergence behavior as shown in Figure S14 e-f. This criterion ensured that the numerical solutions reached a converged state before post-processing.

2.2 Numerical simulation results of the paperflake.

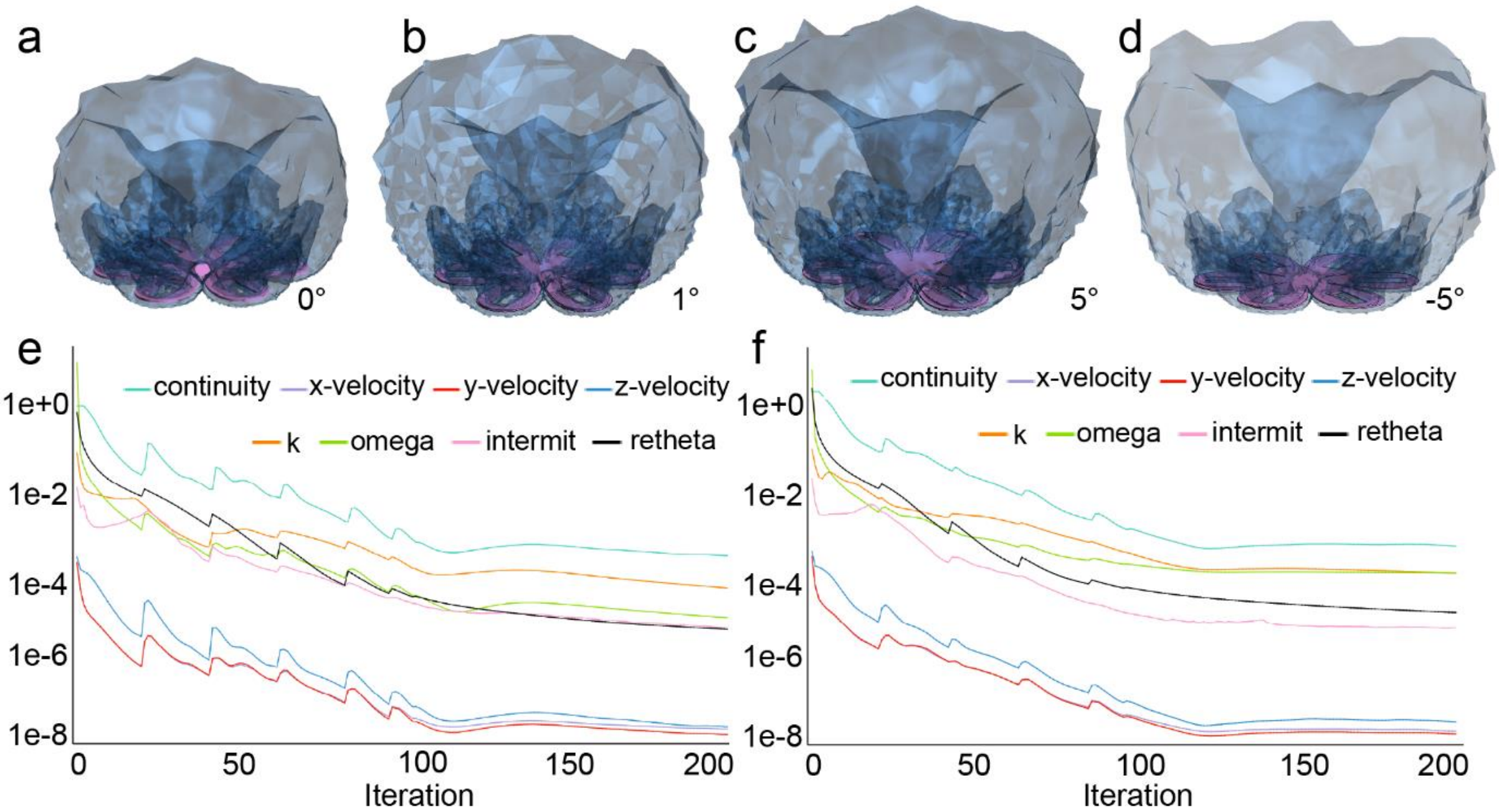

Figure S14. CFD analysis of flow structures and numerical convergence. a–d) Iso-surfaces of Q-criterion ($Q = 50$) for paperflake pairs with $\alpha = 0°$, $1°$, $5°$and $-5°$, $L = 0$. e,f) Evolution of residuals and monitored flow variables during the iterative solution process for $\alpha = 0°$and $1°, L = 0$.

2.2 Numerical simulation result of the paperflake.

Since contour plots can only be visualized in the plane configuration, the 3D simulation results are presented in the form of streamlines. Figure S15 and S16 present the pressure distribution and wind velocity field around a single paperflake, respectively. The pressure distribution and wind velocity field in a paperflake pair are exemplified in Figure S17 and S18.

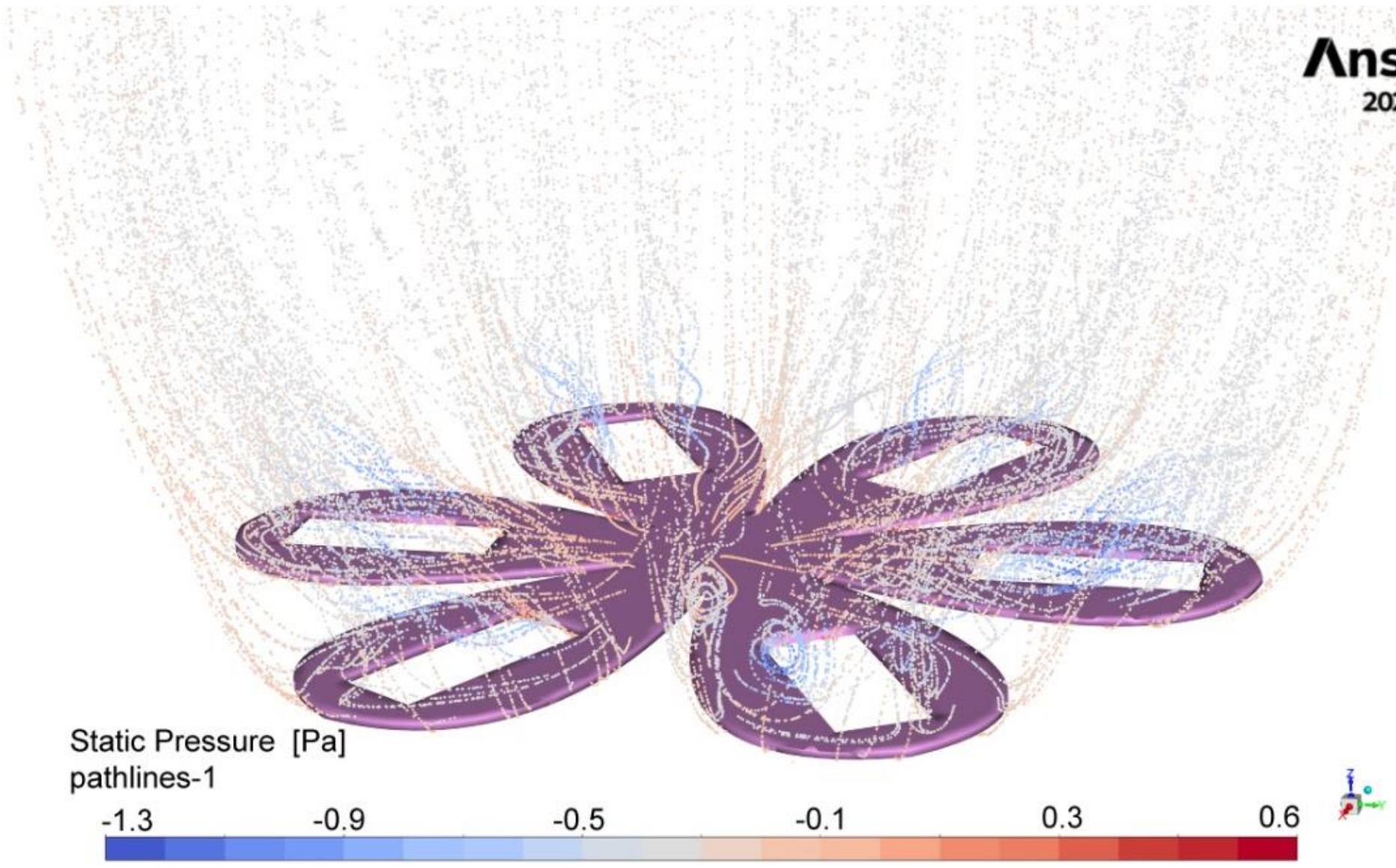

Figure S15. Three-dimensional pressure distribution in the downstream region of a single paperflake.

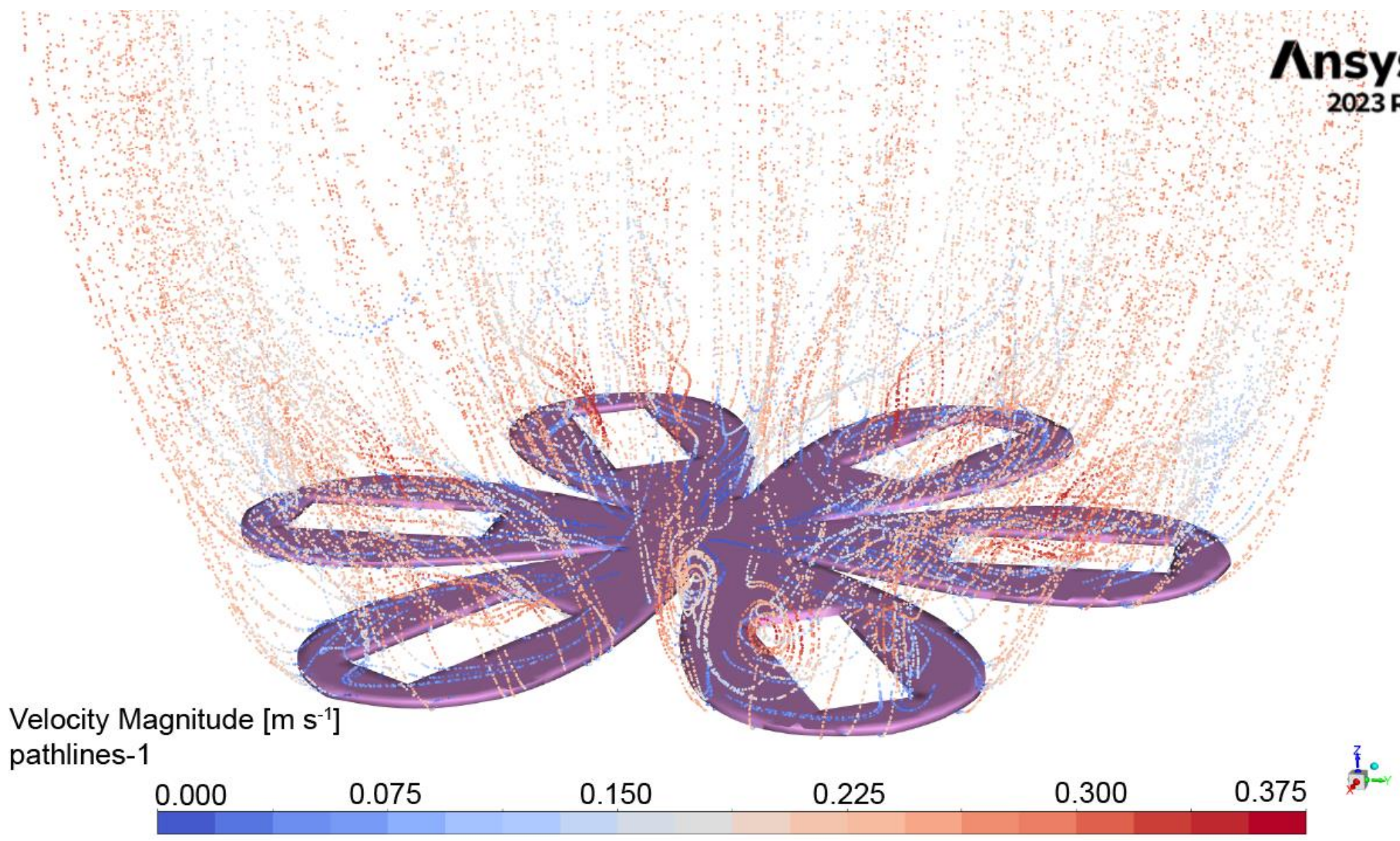

Figure S16. Three-dimensional velocity field distribution in the downstream region of a single paperflake.

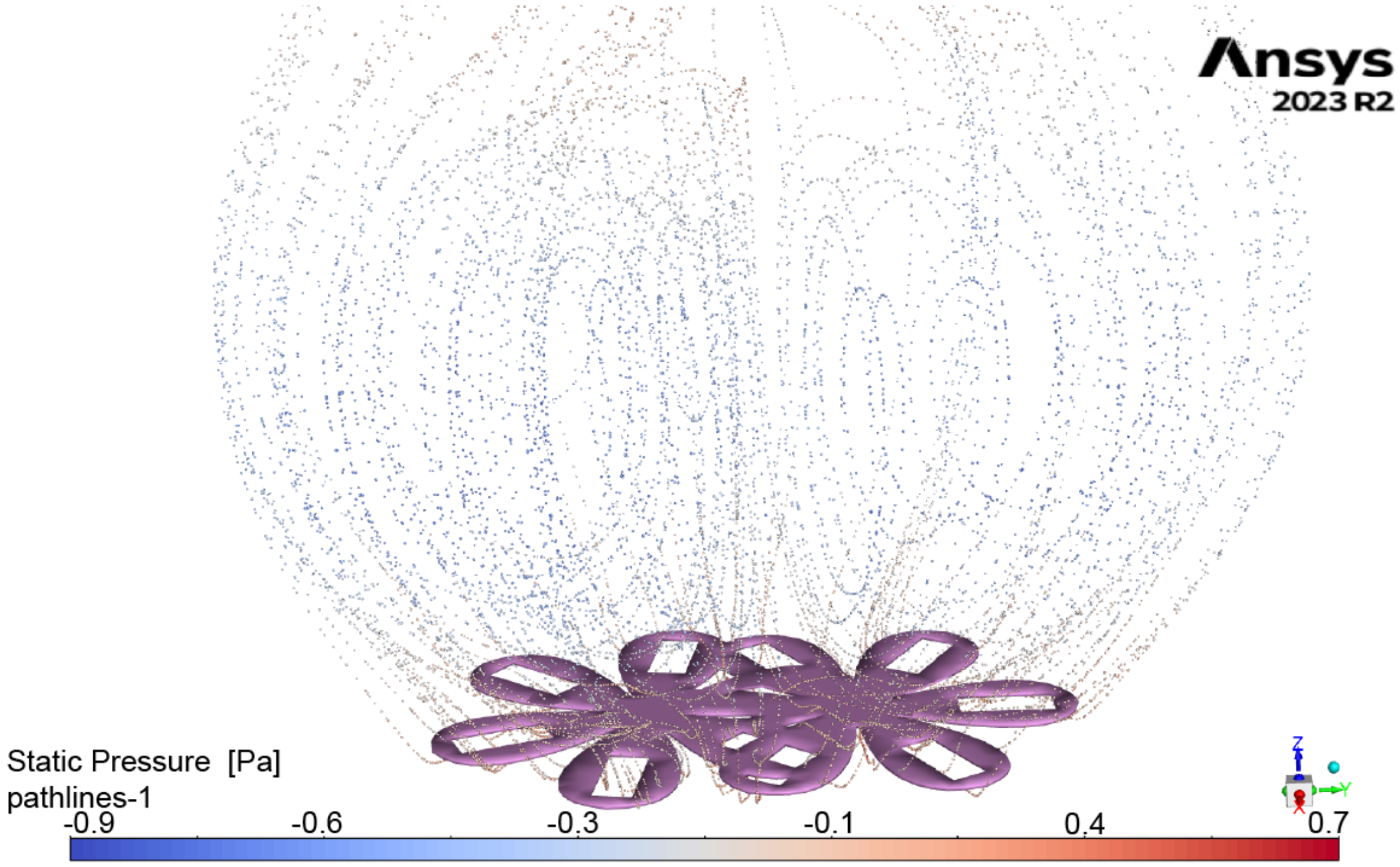

Figure S17. Three-dimensional pressure distribution in the downstream region of a paperflake pair. Clustering parameters: $\alpha = 20°$, $L = 12$ mm.

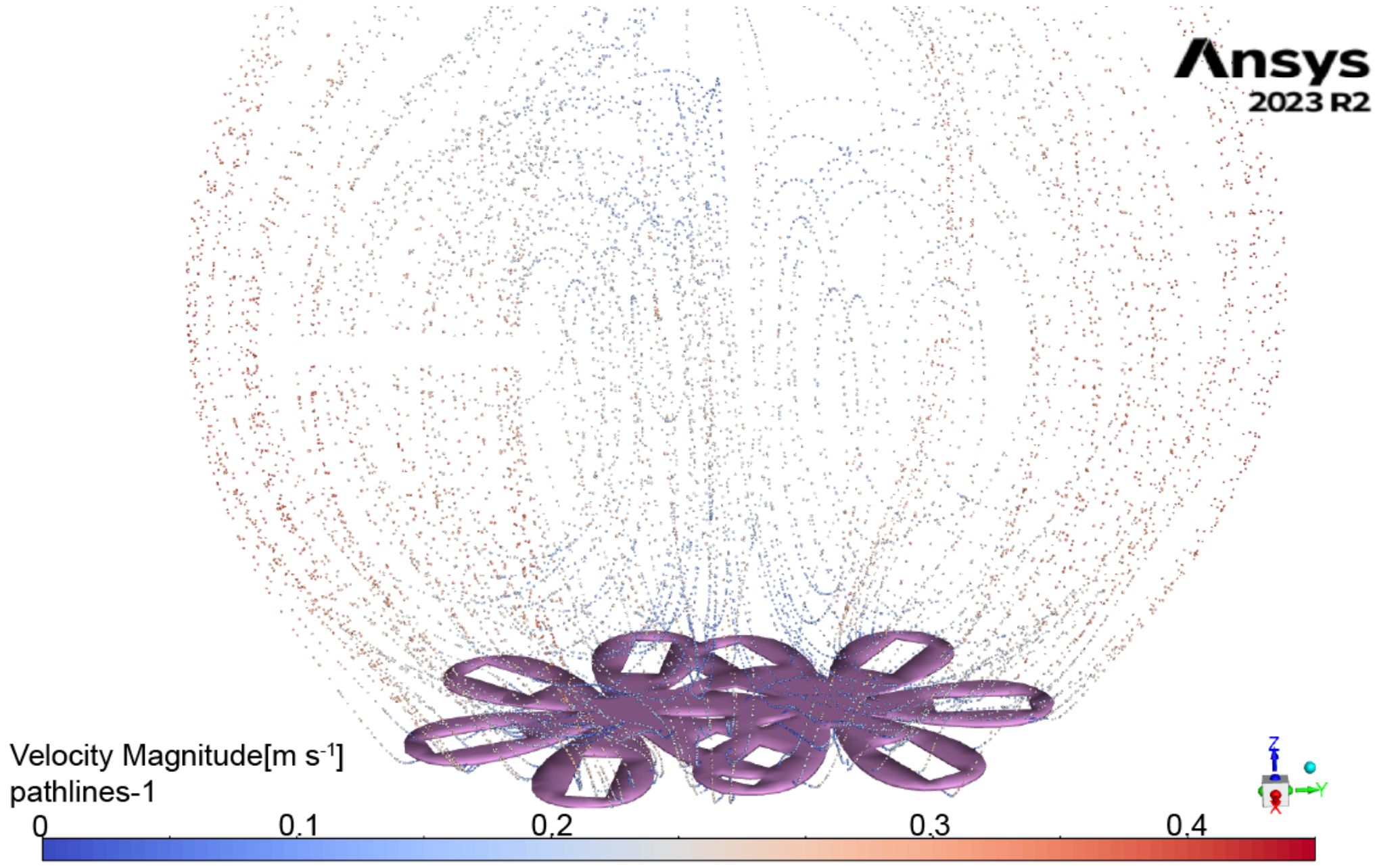

Figure S18. Three-dimensional velocity field distribution in the downstream region of a paperflake pair. Clustering parameters: $\alpha = 20°$, $L = 12$ mm.

When the fluid pressure field around a mid-air object body is inhomogeneously distributed, the object would experience different magnitudes of pressure at different parts of

the body. Torque is produced through the sum of all forces acting on the body surface. Mathematically, this can be expressed as, $\mathbf{M} = \sum_i \boldsymbol{r}_i \times \mathbf{F}_i$, where $\mathbf{M}$ is the torque, $\boldsymbol{r}_i$ is the vector from the centroid to the point where the force acts, $\mathbf{F}_i$ is the force acting at that point. Only if the pressure field is homogeneously distributed around the object, or net force line passes through the centroid of the object, the torques cancel out and the body does not rotate. Due to the complexity of aerodynamics, the simulation was limited to transient analysis of the flow around a static structure.

3. References

[1] Menter, Florian R., Robin Blair Langtry, S. R. Likki, Y. Bora Suzen, P. G. Huang, and S. Völker. "A correlation-based transition model using local variables—Part I: Model formulation." *Journal of turbomachinery* 128, no. 3 (2006): 413-422.

4. Captions for Supplementary Movies S1-5

Movie S1. Collective motions of paperflakes in wind tunnel. The video is played in real time.

Movie S2. Geometry-determined gliding. Top-view recordings of paperflake-pairs at a fixed relative angle of 5° with varying separation distances ($L = 0,\ 4,\ 8$ mm). The video is played at 0.25x speed.

Movie S3. Geometry-determined spinning. Top-view recordings of paperflake-pairs at zero separation distance ($L = 0$ mm) with varying relative angles. The video is played at 0.25x speed.

Movie S4. Collective motions of paperflakes in the wind tunnel at low Reynolds numbers. The video is played in real time.

Movie S5. Multi-view recordings of a paperflake-pair in the wind tunnel. The video is played at 0.25× speed.
